# A meta‐analysis of the effect of visiting zoos and aquariums on visitors’ conservation knowledge, beliefs, and behavior

**DOI:** 10.1111/cobi.14237

**Published:** 2024-02-02

**Authors:** Xavier McNally, Thomas L. Webb, Charlotte Smith, Andrew Moss, Jilly Gibson‐Miller

**Affiliations:** ^1^ ICOSS building University of Sheffield Sheffield UK; ^2^ Cedar House Chester Zoo Chester UK; ^3^ The Wave University of Sheffield Sheffield UK

**Keywords:** aquariums, behavior, behavioral science, beliefs, meta‐analysis, systematic review, zoos, acuarios, ciencias del comportamiento, comportamiento, creencias, metaanálisis, revisión sistemática, zoológicos

## Abstract

Zoos and aquariums are well placed to connect visitors with the issues facing biodiversity globally and many deliver interventions that seek to influence visitors’ beliefs and behaviors with respect to conservation. However, despite primary studies evaluating the effect of such interventions, the overall effect of engaging with zoos and the factors that influence this effect remain unclear. We conducted a systematic review to investigate the effect of zoo‐led interventions on knowledge, beliefs (attitudes, intentions, self‐efficacy, and social norms), and behavior among zoo visitors. These outcomes were identified using the Theory of Planned Behavior as a theoretical lens. We identified and described the nature of zoo‐led interventions in 56 studies and used the behavior change technique (BCT) taxonomy to identify 6 specific BCTs used in interventions to date. Multilevel meta‐analyses revealed a small to medium positive effect of engaging with zoo‐led interventions on outcomes (*d*_+_ = 0.40, 95% confidence interval = 0.28–0.51). Specifically, visitors were more knowledgeable about conservation issues, held more favorable attitudes toward conservation, and reported being more likely to act for the benefit of biodiversity. No evidence of publication bias was present. Effect sizes were, however, heterogeneous and subgroup analyses revealed that the nature of the intervention or type of outcome did not explain this variance. Larger effects were, however, found in studies conducted at a single institution relative to research at multiple institutions and studies that used within‐participant designs relative to between‐participant designs. Taken together, these findings demonstrate how behavior change frameworks can be used to describe zoo‐led interventions and supports the assertion that zoos and aquariums can promote changes in beliefs and behaviors that may help protect biodiversity.

## Abstract (es)

Metaanálisis del efecto de la visita a acuarios y zoológicos sobre el conocimiento, creencias y comportamientos de conservación de los visitantes © 2024 by John Wiley & Sons, Inc.

**Resumen:**

Los zoológicos y los acuarios están bien posicionados para conectar a los visitantes con temas mundiales de biodiversidad y varios cuentan con intervenciones que buscan influir las creencias y el comportamiento de los visitantes con respecto a la conservación. Sin embargo, con todo y los estudios primarios que evalúan el efecto de dichas intervenciones, aún no está claro el efecto general de participar en los zoológicos y los factores que influyen sobre este efecto. Realizamos una revisión sistemática para investigar el efecto de las intervenciones en los zoológicos sobre el conocimiento, creencias (actitud, intención, autosuficiencia y normas sociales) y comportamiento de sus visitantes. Usamos la teoría del comportamiento planeado como lente teórico para identificar los resultados. Identificamos y describimos la naturaleza de las intervenciones en los zoológicos en 56 estudios y usamos la taxonomía de las técnicas de modificación de conducta (TMC) para identificar seis TMC específicas que se usan hoy en día en dichas intervenciones. Los metaanálisis multinivel revelaron un efecto positivo de pequeño a mediano sobre los resultados causado por la participación en las intervenciones de los zoológicos (*d_+_* = 0.40, 95% CI = 0.28 – 0.51). En específico, los visitantes fueron más conocedores sobre los temas de conservación, tuvieron actitudes más favorables hacia la conservación y reportaron tener mayor probabilidad de actuar en beneficio de la biodiversidad. No hubo evidencias de sesgos en las publicaciones. Sin embargo, el tamaño de los efectos fue heterogéneo y el análisis de los subgrupos reveló que la naturaleza de la intervención o el tipo de resultados no explicaban esta varianza. A pesar de esto, encontramos efectos más grandes en los estudios realizados en una sola institución en relación con la investigación realizada en varias instituciones y los estudios que usaron diseños intraparticipantes en relación a los que usaron diseños interparticipantes. Nuestra revisión demuestra cómo los marcos de modificación conductual pueden usarse para describir las intervenciones en los zoológicos y acuarios y respalda la aseveración de que estas instituciones pueden promover cambios en las creencias y el comportamiento que pueden ayudar a proteger la biodiversidad.

## INTRODUCTION

It is widely accepted that human actions are a primary cause of environmental degradation globally. For instance, rising demands for material consumption are resulting in increased extraction of natural resources and habitat loss (Dietz, 2017; IPBES, 2019; Tilman & Clark, 2014). Zoos and aquariums (hereafter zoos) are well placed to shape visitors’ perspectives of conservation issues (Fraser & Wharton, 2007; Thomas, 2020). Having fewer experiences with the natural world can negatively affect people's likelihood to act for the benefit of biodiversity (Lengieza et al., 2023; Soga & Gaston, 2016). Visiting zoos and engaging with zoo‐led interventions may provide an avenue for people to connect with conservation issues and compensate for the lack of experiences people may have with nature (Barragan‐Jason et al., 2021; Conway, 2011; Rose & Riley, 2022). Specifically, visiting zoos provides a space for informal learning and may increase visitors’ knowledge of conservation issues, improve people's attitudes toward conservation, and drive environmentally sustainable behavior (Ballantyne et al., 2011; Godinez & Fernandez, 2019). However, despite numerous research studies, the impact of a zoo visit on visitors’ beliefs about conservation and behavior is currently unclear, and uncertainty remains surrounding what factors influence this effect (Greenwell et al., 2023; Miranda et al., 2023).

Zoos face increasing pressure to demonstrate measurable impacts on their visitors and society (RSPCA, 2006; Spooner et al., 2023). Clarity is needed on how visiting and engaging with zoos may influence their visitors and how best to demonstrate their contributions to global initiatives. Notable initiatives include the Global Biodiversity Framework from COP15, specifically target 16, which concerns sustainable consumption choices and halving food waste (Moss et al., 2023), and Aichi Biodiversity target 1, which specifies the need to raise awareness about biodiversity values and steps to “use it sustainably” (Moss et al., 2015). The World Association of Zoos and Aquariums (WAZA) has laid out strategic directions for its member institutions. They define conservation as “securing populations of species in natural habitats for the long term” and see the role of zoos as pivotal in reshaping conservation attitudes and behaviors among visitors (Barongi et al., 2015, p. 12). As visitors engage with zoos, their consequent conservation behavior is defined as actions taken to secure populations of species in natural habitats for the long term. To effectively assess the impact of engaging with interventions in zoos on visitors’ conservation behavior, researchers need a better understanding of the beliefs that drive people's conservation behavior. The WAZA conservation strategy, for example, recognizes that zoos need to determine “what motivates people to act” (Barongi et al., 2015, p. 45). We examined the impact of zoo‐led interventions through theoretical frameworks from behavioral science that allow researchers to measure valid outcomes that are likely to predict behavior (e.g., intentions) and describe the nature of the interventions that seek to change these beliefs (Michie et al., 2011).

### Need for a systematic review with meta‐analysis

Systematic approaches have been used to evaluate research conducted in zoos and identify relevant research studies. For example, Mellish et al. (2019) conducted a systematic review that outlines the research methods used in the field, and Schilbert and Scheersoi (2023) conducted a systematic review to summarize the outcomes measured in the research. However, to date, a meta‐analysis has not been conducted to quantitatively estimate the direction and magnitude of the effect of visiting zoos on visitor outcomes. Findings from a meta‐analysis, such as ours, could help zoos determine their impacts and communicate their role in society (Spooner et al., 2023).

### The nature of zoo‐led interventions

Visitors to zoos can engage with conservation issues they would otherwise not engage with and could thus become more likely to carry out actions in support of conservation, such as shifting their consumer choices to more sustainable products (Godinez & Fernandez, 2019). Zoos typically attempt to highlight conservation issues throughout the visitor experience, and they employ a variety of interventions designed to educate visitors and shape their perspectives (Anderson, 1991; Hancock, 2001; Heimlich & Ardoin, 2008). The impact of engaging with interventions offered to zoo visitors has been studied, including the impact of animal feeding demonstrations (Kleespies et al., 2020) and changes to exhibit design on attitudes of zoo visitors (Chiew et al., 2019). A review of research evaluating zoo‐led interventions describes the types of interventions implemented by zoos, concluding that most studies evaluated the zoo visit as a whole and those that describe a specific intervention tended to evaluate presentations from zoo staff or signage (Mellish et al., 2019). Research that includes accurate and replicable descriptions of the interventions implemented can be replicated more easily. However, currently there is no standardized methodology for designing, describing, or evaluating interventions in zoo‐led research.

Behavioral science has made significant advances in describing the nature of interventions in other contexts, such as interventions in health and clinical psychology (Hilton & Johnston, 2017; Ogden, 2019), and in sustainability research, such as tackling plastic waste (Allison et al., 2022). These frameworks might therefore be translated to describe the types of engagements zoo visitors have with interventions in zoos. The behavior change technique (BCT) taxonomy 1, for example, is part of the behavior change wheel method for characterizing and designing behavior change interventions (Michie et al., 2011). The taxonomy describes 93 specific strategies used in interventions seeking to influence behavior, including social support and giving feedback (Michie et al., 2013). The structured nature of the BCT taxonomy provides a means to describe the specific methods by which engaging with zoos may influence visitors’ beliefs and behavior and allows for the systematic accumulation of evidence.

We identified strategies used in zoo‐led interventions with the BCT taxonomy. For example, a conservation campaign evaluated in an Australian zoo aimed to educate visitors about the impact of unsustainable palm oil production and raise public awareness of solutions, such as shifting consumer choices (Pearson, Lowry et al., 2014). The intervention sought to provide targeted knowledge about the potential impact of a behavior (i.e., BCT 5.3: information about social and environmental consequences), communicate information to the target population from a trusted source (i.e., BCT 9.1: credible source), and remove barriers to action by giving people the tools they need to carry out a specific action (i.e., BCT 12.5: adding objects to the environment). By mapping the individual components of zoo‐led interventions (e.g., specific BCTs, context, mode of delivery), researchers can identify what works, when, and for whom.

### Measuring outcomes of zoo‐led interventions

Previous research evaluating the impact of visiting zoos has focused predominantly on changes in knowledge or attitudes (Mellish et al., 2019; Schilbert & Scheersoi, 2023). This mirrors findings discussed in conservation education research more generally (Heimlich & Ardoin, 2008; Nilsson et al., 2019). However, the link between knowledge of environmental issues and behavior is relatively weak (Heeren et al., 2016; McGuire, 2015), leading people to refer to a knowledge–action gap that must be bridged (Knutti, 2019). Therefore, although advancing knowledge is important, conceptual and methodological insights from behavioral science may provide a way to measure and better predict the likelihood that people will act more sustainably after engaging with conservation education (Balmford et al., 2021; Mascia et al., 2003; Nielsen et al., 2021; Saunders et al., 2006).

We identified beliefs likely to shape people's behavior, specifically conservation behavior, with the Theory of Planned Behavior (TPB) (Ajzen, 1991). According to the TPB, the most proximal determinant (or immediate precursor) of behavior is the intention to perform the behavior in question (Ajzen, 2020). Intentions, in turn, are determined by an individuals’ positive versus negative evaluations of the behavior (i.e., attitudes), their perception of social approval from important others (i.e., subjective norms), and their perception of the ease or difficulty of performing the behavior (i.e., perceived behavioral control). We focused on self‐efficacy as a measure of zoo visitors’ beliefs in their capability to perform conservation behaviors because perceived behavioral control relies on additional information about external factors, such as the availability of opportunities (Ajzen, 1991; Ajzen & Timko, 1986; Bandura, 1997). The TPB has been extensively used as a framework for understanding behavior, and evidence suggests components of the theory can reliably predict behavior (Armitage & Conner, 2001).

**FIGURE 1:**
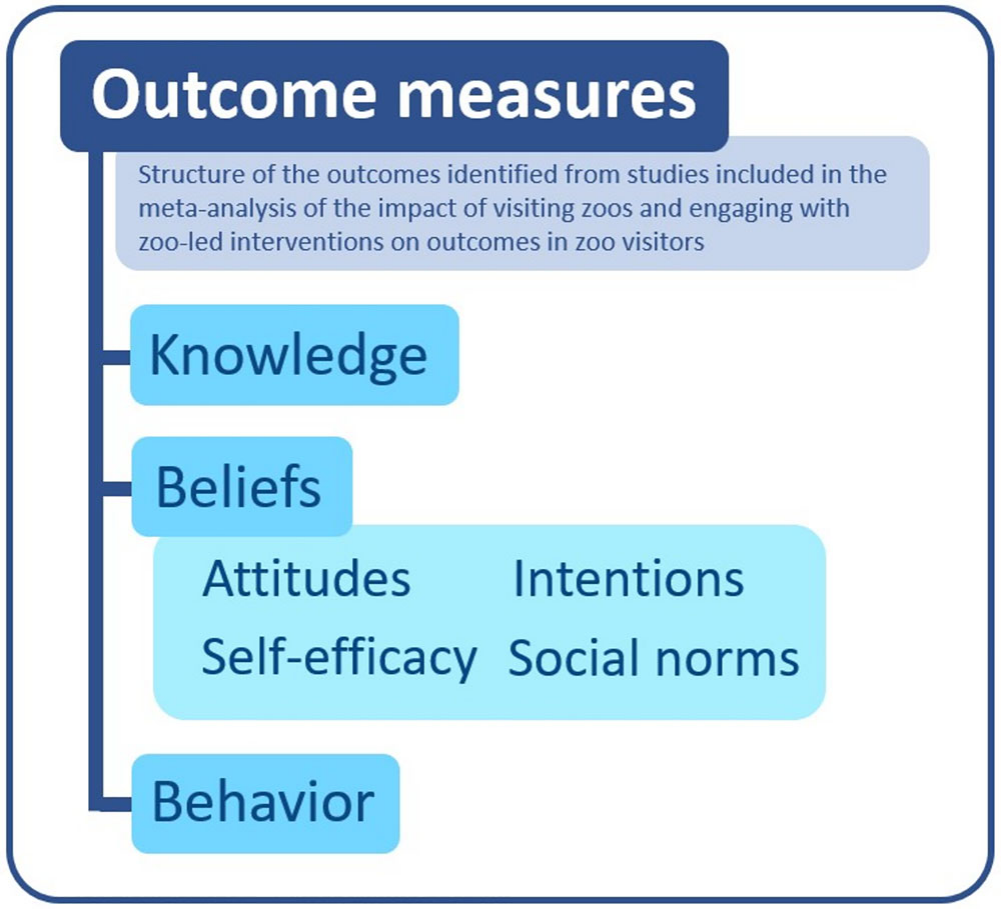
Structure of the outcomes identified from studies included in the meta‐analysis of the impact of visiting zoos and engaging with zoo‐led interventions on outcomes in zoo visitors.

The beliefs about conservation we assessed are, therefore, attitudes (i.e., people's evaluation of a behavior and its consequences), intentions, perceived behavioral control beliefs described as self‐efficacy (i.e., people's beliefs in their ability to carry out a particular behavior), and people's perceptions of subjective norms. Finally, we assessed behavior and knowledge, which is a commonly used measure (Mellish et al., 2019; Nygren & Ojalammi, 2018; Schilbert & Scheersoi, 2023). We included measures concerning knowledge of conservation issues, their solutions, and biodiversity knowledge (i.e., species biology and ecology) (Figure 1).

Our primary aim was to estimate the effect of visiting zoos and engaging with different zoo‐led interventions on visitor outcomes concerning conservation. We identified the nature of the zoo visit by describing the zoo‐led interventions evaluated (i.e., the independent variable); described the outcomes being evaluated using the TPB (i.e., the dependent variable); and identified whether and how the nature of the intervention affects these outcomes. The inclusion criteria for the review were intentionally broad to capture the diversity in zoo‐led interventions and the factors that may affect the impact of zoo‐led interventions, such as study design and the sample characteristics. As such, we asked what effect does a zoo visit or engagement with zoo‐led interventions have on visitors’ beliefs about conservation and behavior, their knowledge, and their behavior and what factors moderate this impact?

## METHODS

The protocol for the review was preregistered on the Open Science Framework (OSF) (accessed via https://osf.io/dtz6q/).

### Inclusion and exclusion criteria

Included studies had to have evaluated the impact of engaging with interventions that were led by zoos or aquariums. Studies investigating the effects of education programs delivered outside the zoo or delivered by school staff were excluded (e.g., Kleespies et al., 2021; Moss et al., 2017; Pearson, Mellish et al., 2014). Studies that asked participants to retrospectively recall whether they had visited a zoo (e.g., Mulder et al., 2009; Taylor & Duram, 2021) were not included because we did not consider them a direct evaluation of a zoo‐led intervention. Studies had to have measured one or more of the defined outcomes in response to a zoo visit or zoo‐led intervention (Figure 1). This included the evaluation of knowledge, beliefs (e.g., attitudes, social norms, intentions, self‐efficacy), and behavioral outcomes concerning conservation. Studies that measured outcomes unrelated to conservation (e.g., customer satisfaction, perceptions of animal welfare) were excluded (e.g., Godinez et al., 2013; Marshall et al., 2019). Included studies evaluated the impact of interventions on zoo visitors; therefore, studies evaluating outcomes related to zoo staff or participants sourced through online channels (e.g., Survey Monkey) were excluded (e.g., Jiang et al., 2006; Khalil et al., 2017; Pearson, Mellish et al., 2014).

### Search strategy

Primary studies were identified using search terms reflecting 3 filters: (A) zoo or aquariums, (B) outcomes, and (C) visit or engagement. We used the search terms (Appendix S1) to search the online database Web of Science to identify peer‐reviewed articles in November 2021. The search had no language, time, or geographical constraints applied and captured the following editions in the Web of Science database (A&HCI, BKCI‐SSH, BKCI‐S, CCR‐EXPANDED, ESCI, IC, CPCI‐SSH, CPCI‐S, SCI‐EXPANDED, and SSCI). To identify unpublished research, we conducted a search in ProQuest Global Thesis and Dissertations in November 2021. Gray literature sources in the field, including *IZE Journal* (from 2017 to 2021) and *WAZA Magazine* (from 2009 to 2021), were accessed in November 2021 and searched manually.

### Screening and eligibility

**FIGURE 2:**
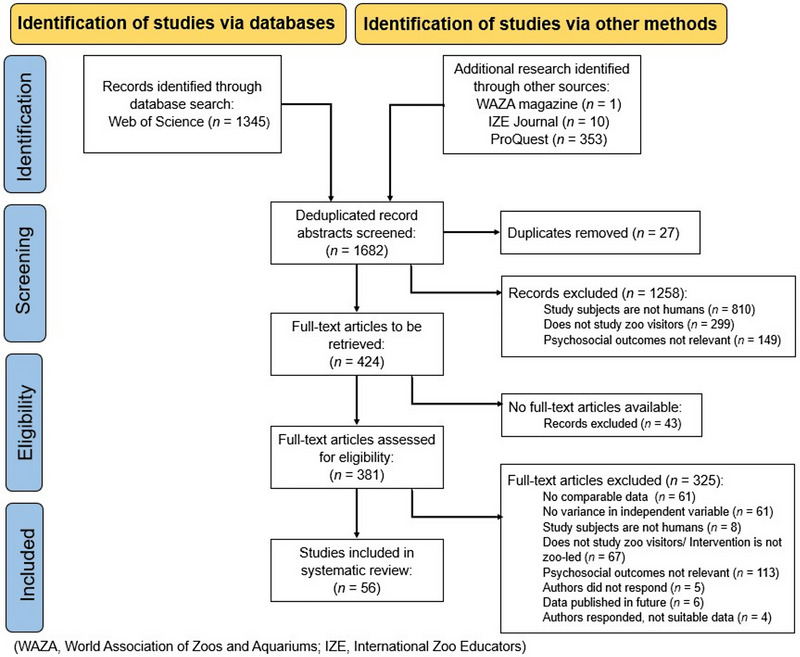
Screening process of studies identified via databases and other methods for the meta‐analysis of the impact of visiting zoos and engaging with zoo‐led interventions on outcomes in zoo visitors.

The screening process was reported using PRISMA guidelines (Page et al., 2020) (Figure 2). At each stage, the reasons for exclusion were tracked with the numbers of studies falling into each category. We stored records identified by the search strategy in an EndNote X9 Library. Initially, duplicates were identified automatically by the software, and then X.M. checked the results manually.

Titles and abstracts were screened by X.M. to assess whether research articles were likely to meet the inclusion criteria. Full texts were retrieved, where possible, for studies included at this stage. Next, retrieved full‐text articles were screened by X.M., with reference to the inclusion criteria. T.L.W. screened a subset (10%) of the full‐text articles, and interrater reliability was assessed using Cohen's kappa (McHugh, 2012). We found 87% agreement (*k* = 0.71) prior to discussions. Inclusion criteria were then augmented to clarify decisions made by the raters following disagreements. For example, research evaluating a virtual tour was excluded because the tour was designed based on the researcher's interpretation of signage around the aquarium, with no input from zoo practitioners (e.g., Jiang et al., 2006).

### Coding

Prior to gathering information from the studies, we designed a data extraction form (Appendix S2) and piloted the form with a subset of studies. The form included definitions of variables and features to be extracted from the primary studies for analysis. A coding manual (Appendix S3) was written to guide researchers when extracting information (e.g., the ways in which outcomes should be classified).

We coded the type of intervention delivered by the primary studies with reference to 9 types of intervention (Table 1) described in a previous systematic review (Mellish et al., 2019). We coded the specific BCTs included in the interventions delivered by the primary studies based on the BCT taxonomy 1 (Michie et al., 2013). The process involved identifying BCTs in the intervention descriptions and including a direct quotation as evidence, as suggested by Michie et al. (2013).

**TABLE 1:** Types of zoo‐led interventions and examples used in the final subset of studies (*n* = 56) in a meta‐analysis of the impact of visiting zoos and engaging with zoo‐led interventions on outcomes in zoo visitors.

Intervention type	Definition of intervention	Example
Digital	Use of online digital media and technologies to engage visitors	Post‐visit information packs, mobile applications
Exhibit design	Comparison of different types of exhibits or experiences	Naturalistic/traditional exhibit design, immersive experiences
Formal education	Zoo‐led education programs to engage visitors	Students exposed to education programs
General school field trip^a^	Zoo experience for school children without formal education	Students not exposed to education programs
General visit	Zoo experience without a specific intervention type described	Entering or exiting visitors evaluated
Keeper talk	Presentation delivered by zoo staff without live animal demonstrations	Talk, presentation, theater, or tour
Live animal interaction	Presentation using planned or live animal demonstrations	Interaction with animals, feeding, or live performance
Multimedia	Use of multimedia technologies in the zoo	Touchscreen, video presentation, interactive elements
Signage	Use of information displayed on various types of medium	Graphics, banners, maps, posters, or in exhibits
Other	Intervention type does not meet the above descriptions	

^a^ Evaluated a school sample, but no specific type of intervention was described.

We classified beliefs about conservation as reflecting changes in visitors’ attitudes toward conservation, self‐efficacy with respect to conservation behaviors, social norms surrounding conservation behaviors, or intentions to act in ways that conserve biodiversity. Outcomes reflecting knowledge were also extracted, including biological knowledge and understanding of conservation issues and relevant behaviors. Finally, behavior was measured in 2 ways: survey responses capturing self‐reported participation in the behavior or recording participation in measurable conservation actions.

The design of the primary studies was coded according to classifications described by the Effective Public Health Practice Project quality assessment tool (EPHPP) (Armijo‐Olivo et al., 2012; Thomas et al., 2004). This tool was designed to assess the quality of quantitative studies in public health practice. However, we did not explicitly code the methodological quality of studies because a previous systematic review assessing research in the zoo context showed that over 80% of the quantitative methodologies were categorized as “weak” according to the EPHPP tool (Mellish et al., 2019). Further to this, risk of bias assessment tools rely on exhaustive information being available in the research and for protocols to be written with this type of interpretation in mind (Chiocchia et al., 2021; Sterne et al., 2019).

We planned to code the use of theory in the primary studies (e.g., to develop the intervention or identify relevant outcomes) using the theory coding scheme developed by Michie and Prestwich (2010). However, our scoping review indicated that reports of the primary studies in this context did not typically describe how theory was used. Therefore, we only coded whether the primary studies mentioned theory.

### Data extraction

X.M. used the data extraction form to extract information from the full‐text articles, and T.L.W. used the data extraction form independently to extract the information from a subset of the full‐text articles. Disagreements were resolved through discussion. If agreement was not reached, further discussion with C.S., A.M., and J.G.M. continued until a consensus was reached. The level of agreement was high (88%) prior to the discussion of disagreements. Cohen's kappa showed moderate agreement when coding the nature of the intervention (*k* = 0.76) and near perfect agreement when coding the nature of the outcome measures (*k* = 0.96) (McHugh, 2012). These statistics were calculated prior to discussions.

### Effect size calculations

In each of the primary studies, the standardized difference in outcomes as a function of exposure to a zoo‐led intervention was extracted using Cohen's *d* effect size estimates. The majority of studies (82%) only measured outcomes immediately following the intervention. Therefore, for the minority of studies that reported outcomes at multiple points, the effect sizes were computed using data from the shortest follow‐up point in an effort to permit comparison between the effects of different interventions. The average time between the intervention and measurement of outcomes across the primary studies was 0.50 weeks (SD 2.15, range 0–12 weeks).

We extracted effect sizes reported in the primary studies, if available (*n* = 6). If effect sizes were not reported, then X.M. calculated effect sizes from the data and test statistics reported (*n* = 50). If sufficient information to calculate effect sizes was not available, then we contacted authors to ask for further information.

We calculated effect sizes with online tools provided by Psychometrica (Lenhard & Lenhard, 2016) or a formula described by Lakens (2013). We extracted the difference in the outcome or outcomes between the intervention condition and comparison condition in studies that had between‐participant designs (e.g., the research evaluated visitors entering an exhibit and compared them with different visitors exiting the exhibit [Smart et al., 2021]). We extracted the difference between responses before and after an intervention in studies that used within‐participant designs (e.g., the research evaluated visitors’ connection to nature before and after guided tours [Kleespies et al., 2020]). We used different formulas to calculate the variance for Cohen's *d* in between‐participant designs (Borenstein et al., 2009; Cooper et al., 2009) and within‐participant designs (Borenstein et al., 2009; Gibbons et al., 1993) to account for correlations between the 2 samples in within‐participant designs. Where necessary, we converted standard errors to standard deviations with a formula in the *Cochrane Handbook* (Higgins et al., 2022, chapter 6.5.2.2). Details of the methods we used to calculate effect sizes are in Appendix S3.

### Multilevel meta‐analysis

The meta‐analysis was carried out in R Studio, and the following packages were used to conduct the primary analyses, generate outputs, and conduct further analyses: metafor (Viechtbauer, 2010), ggplot2 (Wickham, 2016), tidyverse (Wickham et al., 2019), janitor (Firke, 2021), clubSandwich (Pustejovsky, 2022), and dmetar (Harrer et al., 2019). The code is in Appendix S4.

Some of the primary studies measured more than one outcome, for example, knowledge, attitudes, self‐efficacy, intentions, and behavior (Clayton et al., 2017). Therefore, to maintain the independence of effect sizes and the validity of the meta‐analysis, a multilevel approach was used that accounted for the possibility that effect sizes within a study were correlated (Cheung, 2019; Van den Noorgate et al., 2013). Specifically, a multilevel random effects model with a restricted maximum likelihood (REML) estimator was built to capture dependency structures in the data set. Code was written in RStudio with the rma.mv() function in the metafor package (Viechtbauer, 2010). On each level of the model, pooling occurs that nests effect sizes into clusters (Harrer et al., 2021). The model has 3 levels: (1) considers the sampling variance, (2) identifies the effect sizes clustered in the grouping variable in level 3, and (3) identifies the distinct studies by which to group the effect sizes.

### Multilevel model validation

To validate the use of a multilevel model, we generated a simpler 2‐level model that sets the between‐study heterogeneity to zero. This comparison tests whether nesting effect sizes within studies (i.e., level‐3 model) generated a model that fit the data set more reliably than not doing so (i.e., level‐2 model). An analysis of variance (ANOVA) was used to compare the 2 models. The Akaike information criterion and Bayes information criterion values were lower for the level‐3 model and the likelihood ratio test was significant ($\chi_{1}^{2}$ = 110.68, *p* < 0.001), which suggested that the level‐3 model was a better fit. The weight given to the studies in the model was not skewed in relation to the sampling variance.

### Sensitivity analyses

Outliers were identified using Cook's distance influence analysis, which identifies outlying effect sizes when the 95% confidence intervals (CIs) fall outside the CIs of the overall pooled effect size estimate. Outliers identified in the influence analysis (*n* = 4) are listed in Table 2. We conducted a sensitivity analysis with the outliers removed to evaluate the impact of the outliers on the overall pooled estimate.

**TABLE 2:** Summary of the characteristics of studies included in the meta‐analysis of the impact of visiting zoos and engaging with zoo‐led interventions on outcomes in zoo visitors.

Study	*n*	Type of intervention	Behavior change techniques^a^	Outcome measured	Location of delivery	Region	Source	Study design	Effect measurement	Participants	Data type	Sample
Anderson et al., 2003	260	Live animal interaction	0	Attitudes	Zoo	North America	Web of Science	Controlled comparison	Immediate	Between participant	Quantitative	Adult
	131	Keeper talk	0	Attitudes								
Ballantyne et al., 2018	475	Digital	1.9, 5.3, 9.1	Behavior	Zoo	Multiple	Web of Science	Cohort analytic	Delayed	Between participant	Quantitative	Adult
Bueddefeld & Van Winkle, 2017	236	Digital	5.3, 9.1	Behavior	Zoo	North America	Web of Science	Cohort analytic	Delayed	Between participant	Mixed methods	Adult
Carlin, 1999	97	School field trip	0	Attitudes	Zoo	North America	ProQuest	Cohort	Immediate	Within participant	Mixed methods	School
	97	School field trip	0	Knowledge								
Chalmin‐Pui & Perkins, 2017, ^b^	50	General visit	0	Knowledge	Zoo	Europe	Web of Science	Cohort	Immediate	Within participant	Mixed methods	Adult
Chiew et al., 2019	495	Exhibit design	0	Attitudes	Zoo	Australia	Web of Science	Controlled comparison	Immediate	Between participant	Quantitative	Adult
Chung et al., 2019	79	Digital	0	Knowledge	Zoo	North America	Web of Science	Cohort analytic	Immediate	Between participant	Quantitative	Child
Clayton et al., 2017	172	General visit	0	Attitudes	Zoo	Europe	Web of Science	Cohort	Immediate	Between participant	Quantitative	Adult
	172	General visit	0	Self‐efficacy								
	172	General visit	0	Knowledge								
	172	General visit	0	Intentions								
	172	General visit	0	Behavior								
Clayton et al., 2018	521	Signage	1.9, 5.3	Attitudes	Zoo	Asia	Web of Science	Controlled comparison	Immediate	Between participant	Quantitative	Adult
	510	Signage	1.9, 5.3	Self‐efficacy	Zoo	Asia	Web of Science	Controlled comparison		Between participant	Quantitative	Adult
Collins et al., 2020	110	Formal education	0	Knowledge	Zoo	Europe	Web of Science	Cohort analytic	Immediate	Within participant	Quantitative	Child
Craig & Vick, 2021	302	Keeper talk	5.3, 9.1	Attitudes	Zoo	Europe	Web of Science	Controlled comparison	Immediate	Between participant	Quantitative	Adult
da Silva et al., 2021	72	Live animal interaction	5.3	Attitudes	Zoo	South America	Web of Science	Cohort	Immediate	Within participant	Quantitative	School
Falk & Adelman, 2003	100	General visit	5.3	Attitudes	Aquarium	North America	Web of Science	Cohort	Immediate	Within participant	Mixed methods	Adult
	100	General visit	5.3	Knowledge								
Geiger et al., 2017	6244	Keeper talk	5.3, 9.1	Attitudes	Zoo and aquarium	North America	Web of Science	Cohort	Immediate	Between participant	Quantitative	Adult
	5612	Keeper talk	5.3, 9.1	Knowledge								
	6339	Keeper talk	5.3, 9.1	Intentions								
Herendeen, 2017	367	Exhibit design	0	Attitudes	Zoo	North America	ProQuest	Controlled comparison	Immediate	Between participant	Quantitative	Adult
Jacobson et al., 2017	312	Exhibit design	0	Attitudes	Zoo	North America	Web of Science	Controlled comparison	Immediate	Between participant	Quantitative	Adult
Jensen, 2014	2839	Formal education	0	Knowledge	Zoo	Europe	Web of Science	Cohort analytic	Immediate	Between participant	Mixed methods	School
Kelly & Skibins, 2021	1044	General visit	5.3, 8.1	Attitudes	Zoo	Australia	Web of Science	Cohort	Immediate	Between participant	Quantitative	Adult
	1044	General visit	5.3, 8.1	Self‐efficacy								
	1046	General visit	5.3, 8.1	Intentions								
Kim Ho et al., 2018, ^c^	204	Formal education	0	Attitudes	Other	Asia	IZE Journal	Cohort	Immediate	Within participant	Mixed methods	School
	204	Formal education	0	Knowledge								
Kirchgessner, 2014	169	Live animal interaction	5.3, 9.1	Intentions	Zoo	North America	ProQuest	Cohort	Delayed	Within participant	Quantitative	School
Kleespies et al., 2020	240	Keeper talk	0	Attitudes	Zoo	Europe	Web of Science	Cohort analytic	Immediate	Within participant	Quantitative	School
	368	Live animal interaction		Attitudes								
Lakes, 2016	278	Keeper talk	0	Attitudes	Zoo and aquarium	North America	ProQuest	Controlled comparison	Immediate	Between participant	Quantitative	Adult
	278	Keeper talk	0	Intentions								
Liu, 2017	367	Formal education	0	Knowledge	Zoo	Australia	IZE Journal	Cohort	Immediate	Within participant	Mixed methods	School
Lukas et al., 2014	2000	General visit	0	Attitudes	Zoo	North America	Web of Science	Cohort analytic	Immediate	Between participant	Quantitative	Adult
	2000	General visit	0	Knowledge								
MacDonald, 2015	68	Live animal interaction	1.9, 5.3, 9.1	Knowledge	Zoo	Australia	Web of Science	Cohort analytic	Immediate	Between participant	Quantitative	Adult
	68	Live animal interaction	1.9, 5.3, 9.1	Behavior								
Mallavarapu & Taglialatela, 2019	489	Exhibit design	5.3, 9.1	Attitudes	Zoo	North America	Web of Science	Cohort analytic	Immediate	Between participant	Quantitative	Adult
	489	Exhibit design	5.3, 9.1	Knowledge								
McLeod & Rawson, 2019	258	Live animal interaction	0	Attitudes	Zoo	Australia	IZE Journal	Controlled comparison	Immediate	Between participant	Quantitative	School
Mellish et al., 2017	99	Keeper talk	5.3, 9.1, 12.5	Intentions	Zoo	Australia	Web of Science	Controlled comparison	Immediate	Between participant	Quantitative	Adult
	160	Keeper talk	5.3, 9.1, 12.5	Behavior								
Mellish et al., 2019	374	Live animal interaction	1.9, 5.3, 6.1, 9.1	Attitudes	Zoo	Australia	Web of Science	Cohort analytic	Immediate	Between participant	Mixed methods	Adult
	374	Live animal interaction	1.9, 5.3, 6.1, 9.1	Knowledge								
	374	Live animal interaction	1.9, 5.3, 6.1, 9.1	Intentions								
Miller et al., 2013	793	Live animal interaction	0	Attitudes	Zoo and aquarium	North America	Web of Science	Cohort	Immediate	Within participant	Quantitative	Adult
	793	Live animal interaction	0	Knowledge								
	793	Live animal interaction	0	Intentions								
	793	Live animal interaction	0	Behavior					Delayed			
Miller et al., 2020	80	Live animal interaction	5.3, 9.1	Attitudes	Zoo	North America	Web of Science	Cohort analytic	Immediate	Between participant	Quantitative	Adult
	80	Live animal interaction	5.3, 9.1	Knowledge								
	80	Live animal interaction	5.3, 9.1	Intentions								
Moss et al., 2015	5661	General visit	0	Knowledge	Zoo and aquarium	Multiple	Web of Science	Cohort	Immediate	Within participant	Qualitative	Other^d^
Moss et al., 2017	2743	General visit	0	Knowledge	Zoo and aquarium	Multiple	Web of Science	Cohort	Immediate	Within participant	Qualitative	Other^d^
Pavitt & Moss, 2019	468	Exhibit design	0	Attitudes	Zoo	Europe	Web of Science	Cohort analytic	Immediate	Between participant	Quantitative	Adult
	468	Exhibit design	0	Self‐efficacy								
	468	Exhibit design	0	Intentions								
Pearson, Lowry et al., 2014	195	Signage	1.9, 5.3, 9.1, 12.5	Attitudes	Zoo	Australia	Web of Science	Controlled comparison	Immediate	Between participant	Quantitative	Adult
	195	Signage	1.9, 5.3, 9.1, 12.5	Knowledge								
	195	Signage	1.9, 5.3, 9.1, 12.5	Intentions								
	195	Signage	1.9, 5.3, 9.1, 12.5	Behavior								
Price et al., 1994	149	Exhibit design	0	Knowledge	Zoo	Europe	Web of Science	Controlled comparison	Immediate	Between participant	Mixed methods	Adult
Randall, 2011	475	Formal education	0	Attitudes	Zoo	North America	ProQuest	Controlled comparison	Immediate	Between participant	Quantitative	School
Randler et al., 2012	845	Formal education	0	Knowledge	Zoo	Europe	Web of Science	Cohort analytic	Immediate	Between participant	Quantitative	School
Rato, 2020	817	Formal education	0	Attitudes	Zoo	Europe	ProQuest	Cohort	Immediate	Within participant	Mixed methods	School
	817	Formal education	0	Knowledge								
Roa, 2016	579	General visit	0	Attitudes	Zoo	North America	ProQuest	Cohort	Immediate	Between participant	Quantitative	Adult
	579	General visit	0	Knowledge								
	579	General visit	0	Intentions								
Roberts, 2013, ^b^	358	General visit	5.3	Knowledge	Other	North America	ProQuest	Cohort	Immediate	Between participant	Quantitative	Adult
Sampson et al., 2020	130	Other^e^	0	Attitudes	Zoo	Europe	Web of Science	Cohort	Immediate	Between participant	Mixed methods	Adult
	130	Other^e^	0	Intentions								
Sattler & Bogner, 2017, ^b^	117	Formal education	5.3	Knowledge	Zoo	Europe	Web of Science	Cohort	Immediate	Within participant	Quantitative	School
Sellmann & Bogner, 2013, ^b^	145	Formal education	5.3	Knowledge	Other	Europe	Web of Science	Controlled comparison	Immediate	Between participant	Quantitative	School
Skibins & Powell, 2013	723	General visit	0	Attitudes	Zoo and aquarium	North America	Web of Science	Cohort	Immediate	between participant	Quantitative	Adult
	723	General visit	0	Intentions								
Smart et al., 2021	110	Exhibit design	0	Knowledge	Zoo	Europe	Web of Science	Cohort analytic	Immediate	Between participant	Mixed methods	Adult
Spooner et al., 2019	128	Keeper talk	0	Knowledge	Zoo	Europe	Web of Science	Cohort	Immediate	Between participant	Mixed methods	Adult
	244	Keeper talk	0	Knowledge								Child
Spooner et al., 2021	564	Live animal interaction	5.3, 12.5	Knowledge	Zoo	Europe	Web of Science	Cohort analytic	Immediate	Between participant	Mixed methods	Adult
Staus, 2012	175	Live animal interaction	5.3	Knowledge	Aquarium	North America	ProQuest	Controlled comparison	Immediate	Between participant	Quantitative	Adult
Syrowicz, 2018, ^b^	159	General visit	0	Knowledge	Zoo	South America	IZE Journal	Cohort	Immediate	Within participant	Qualitative	Other^d^
Torpie‐Sweterlitsch, 2015	943	General visit	5.3, 9.1, 12.5	Knowledge	Zoo	North America	ProQuest	Cohort	Immediate	Between participant	Mixed methods	Adult
Visscher et al., 2009	67	Keeper talk	0	Knowledge	Zoo	North America	Web of Science	Controlled comparison	Immediate	Between participant	Quantitative	School
Waller et al., 2012	156	Live animal interaction	0	Attitudes	Zoo	Europe	Web of Science	Controlled comparison	Immediate	Between participant	Quantitative	Adult
	156	Live animal interaction	0	Knowledge								
Walsh, 2015	58	Keeper talk	5.3, 12.5	Knowledge	Aquarium	North America	ProQuest	Controlled comparison	Immediate	Between participant	Qualitative	Adult
Whitehouse et al., 2014	980	Multimedia	0	Attitudes	Zoo	Europe	Web of Science	Cohort analytic	Immediate	Between participant	Quantitative	Adult
	220	Multimedia	0	Knowledge								
Wunschmann et al., 2017, ^b^	23	School field trip	0	Knowledge	Zoo	Europe	Web of Science	Cohort	Immediate	Within participant	Mixed methods	School

^a^ See Table 3.

^b^ Studies identified as outliers in influence analyses.

^c^ Kim Ho et al. (2013), national park; Roberts (2013), nature reserve; Sellmann and Bogner (2013), botanical garden.

^d^ Adult and child mixed sample.

^e^ New species introduction (*Pyrrhocorax pyrrhocorax*).

A second sensitivity analysis was conducted to check that computing effect sizes with data from the shortest follow‐up point (where data from multiple follow‐up points were available) did not significantly influence the calculation of the pooled estimate in the primary analysis. This involved recomputing effect sizes with data from the longest follow‐up point for 8 studies (14%). The average time between the end of the intervention and assessment of outcomes in these studies was 1.83 weeks (SD 4.32, range 0–24 weeks), ultimately ∼12 days compared with ∼3.5 days in the primary analysis.

### Tests for publication bias

A funnel plot was used to visualize the correlation between the effect sizes and the variance in the data for each study. Effect size values were plotted on the *x*‐axis and the variance on the *y*‐axis. An extension of Egger's regression test for multilevel models was used to identify asymmetry in the funnel plot with the rma.mv() function in the metafor package. A further test to assess publication bias was achieved by adding 2 dummy variables representing published and unpublished studies, respectively, as predictors in the model (Assink & Wibbelink, 2016). Trim and fill analyses were omitted (Duval & Tweedie, 2000) because they have limited power in meta‐analyses with dependent effect sizes (Rodgers & Pustejovsky, 2021).

### Subgroup analyses of moderators

We conducted subgroup analyses to test whether characteristics of the studies (e.g., design of the study) or interventions (e.g., inclusion of specific BCTs) moderated the reported effects. Each characteristic was added to the model as a moderator variable to establish whether the grouping levels significantly affected the pooled effect size estimate. Subgroups were required to contain 3 or more studies to be included in the subgroup analysis. For instance, in the model with the type of intervention added as a predictor, 4 levels were omitted from the subgroup analyses because they failed to meet this threshold (i.e., general school field trip [*n* = 2], multimedia [*n* = 1], signage [*n* = 2], and other [*n* = 1]).

We plotted an evidence‐gap map to identify which combinations of outcomes and types of intervention have been evaluated most frequently and where more evidence is needed (Polanin et al., 2022) (Figure 5). The pooled effect size estimates for combinations of factors shown in the plot were calculated in the same way as those in the subgroup analyses (Viechtbauer, 2023). Combinations of the factors that did not meet the threshold of more than 3 studies were not included in the analyses and were plotted in gray.

## RESULTS

### Overview of included studies

The review included 56 primary studies (Table 2; Appendix S5), providing 75 distinct comparisons between types of zoo‐led interventions and 199 effect sizes representing the effect of zoo‐led interventions on outcomes. The primary studies evaluated a range of interventions (Table 2). The most common were general visits (*n* = 12) and live animal interactions (*n* = 12). Multimedia interventions (*n* = 1) and signage (*n* = 2) were less commonly evaluated. The impact of introducing a new species, the red‐billed chough (*Pyrrhocorax pyrrhocorax*), to the zoo was evaluated and classified as other (*n* = 1). The interventions used 6 techniques from the BCT taxonomy 1 (Michie et al., 2013): BCT 1.9 commitment (*n* = 5); BCT 5.3 information about social and environmental consequences (*n* = 22); BCT 6.1 demonstration of the behavior (*n* = 1); BCT 8.1 behavioral practice or rehearsal (*n* = 1); BCT 9.1 credible source (*n* = 12); and BCT 12.5 adding objects to the environment (*n* = 5). In most cases, the studies included no specific BCTs (*n* = 35); 22 studies included at least one BCT from the taxonomy (*n* = 22). Interventions were delivered in a number of different locations: zoos (*n* = 44), aquariums (*n* = 3), a combination of zoos and aquariums (*n* = 6), and locations described as other (*n* = 3) (e.g., national parks, nature reserves, botanical gardens).

In terms of outcomes, studies predominantly measured knowledge (*n* = 37) and attitudes (*n* = 30), but a subset measured intentions (*n* = 14), behavior (*n* = 7), and self‐efficacy (*n* = 4). No studies evaluated the effect of zoo‐led interventions on social norms with respect to conservation (*n* = 0). In most cases, effects were calculated using data from the primary studies collected immediately after visiting a zoo or engaging with a zoo‐led intervention (*n* = 53; 95%). A smaller number of effects were calculated using data from studies collected after a delay or follow‐up (*n* = 4). Follow‐up data were collected in 20% of studies (*n* = 10) at varying time scales ranging from 3 to 24 weeks.

More studies used between‐participant designs (*n* = 40) than within‐participant designs (*n* = 16). A large number of cohort designs were used to evaluate the effect of a single condition at pretest and posttest (*n* = 23), although this included designs with different samples evaluated before and after the intervention (*n* = 9). A smaller number of studies used a cohort analytic design (*n* = 16) that involved multiple conditions in comparisons of pretest and posttest results or that involved controlled comparisons of multiple distinct conditions (*n* = 17). The majority of studies measured outcomes with quantitative measures (*n* = 36; 64%). The method used to capture data was reported in a large number of studies (*n* = 50; 89%) (i.e., the survey items were reported or the interview schedule was described in detail). Around 38% of the studies mentioned theory (*n* = 21).

The primary studies included data from over 13 countries; however, over one half of the studies were carried out in the United States (*n* = 21) or the United Kingdom (*n* = 11). The studies were conducted in 38 institutions around the world. A number of studies were conducted across multiple institutions (*n* = 10); the remainder were conducted at a single institution (*n* = 46). The studies were published or submitted from 1994 to 2021; the most studies were published or submitted in 2017 (*n* = 11). Research was published in journals reflecting a range of disciplines, including psychology, conservation, visitor studies, education, and environmental sustainability. Around 25% of the studies were gathered from 2 sources: *Environmental Education Research* (*n* = 6) and *Zoo Biology* (*n* = 9). Eleven studies were reported in theses: 5 in PhD dissertation, 4 in MS theses (*n* = 4), and one in an MA thesis (*n* = 1) and an EdD thesis (*n* = 1).

### Effect of visiting and engaging with zoos on outcomes

**FIGURE 3:**
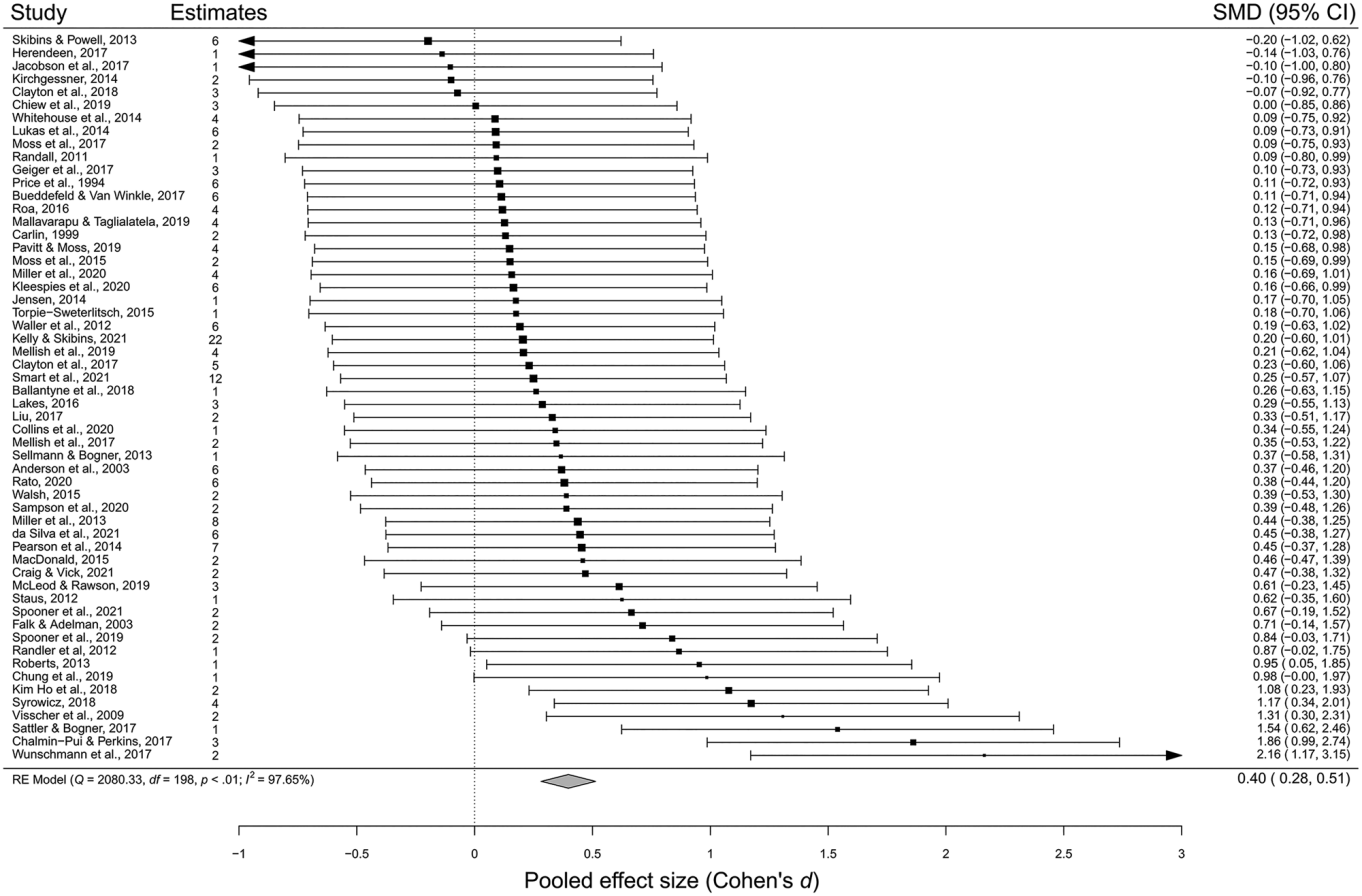
Effect of visiting zoos and engaging with zoo‐led interventions on conservation knowledge, beliefs, and behavior (squares, pooled estimate for each study; error bars, 95% confidence intervals; diamond, overall pooled effect size; SMD, standardized mean difference [Cohen's *d*]).

On average, visiting and engaging with zoos had a small to medium positive effect on visitors’ beliefs about conservation and behavior (Figure 3). The pooled effect size based on the multilevel meta‐analysis was *d*_+_ = 0.40 (95% CI 0.28–0.51, *p* < 0.001). The estimated variance components were *τ* ^2^_level 3_ =  0.17 (95% CI 0.11–0.27) and *τ* ^2^_level 2_ = 0.03 (95% CI 0.02–0.04). This means that 84% of the total variation is attributable to between‐study heterogeneity and 14% to within‐study heterogeneity. Hence, there appeared to be a high degree of variability among estimates from different studies.

### Sensitivity analyses

An analysis of the Cook's distance values identified 4 studies as outliers that may have significantly influenced the calculation of the pooled estimate (Table 2). The pooled effect size based on the multilevel meta‐analysis with these outliers removed was *d*_+_ = 0.30 (95% CI 0.22–0.38, *p* < 0.001); this effect was considered of small to medium magnitude (Cohen, 1992). The estimated variance components were *τ* ^2^_level 3_ =  0.06 (95% CI 0.03–0.11) and *τ* ^2^_level 2_ = 0.03 (95% CI 0.02–0.04). This means that 66% of the total variation was attributable to between‐study heterogeneity and 29% to within‐study heterogeneity. Hence, removing outliers reduced the between‐study variance in effect sizes from 84% in the main analysis to 66%. Given that we had no reason to believe that effect sizes identified as statistical outliers reflected spurious effects that occurred due to error or chance, we retained the outliers in our examination of potential moderators in the hope that we would be able to identify reasons why the effect sizes from these studies differed significantly from those of the majority of studies. Sensitivity analyses with data from the longest period available to calculate the effects showed that the average effect of visiting and engaging with zoos on outcomes assessed was *d*_+_ = 0.38 (95% CI 0.26–0.50, *p* < 0.001).

### Publication bias

**FIGURE 4:**
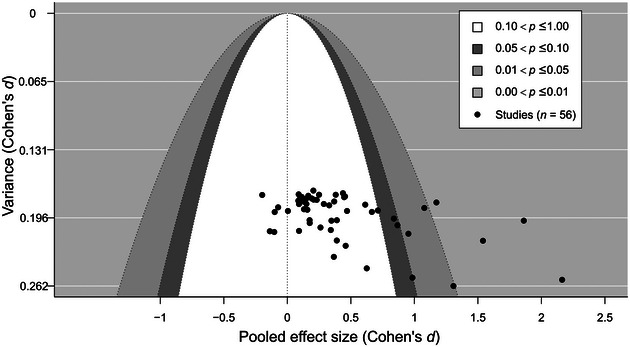
Pooled effect sizes and variances for studies included in the meta‐analysis of the impact of visiting zoos and engaging with zoo‐led interventions on outcomes in zoo visitors.

We found that the effect sizes and the variances were significantly correlated because Egger's test was significant (*t*[197] = 2.17, *p* < 0.05) and the funnel plot was asymmetrical (Figure 4). Publication status did not significantly affect the pooled effect size estimate when published and unpublished studies were compared in a moderator analysis (*p* > 0.05) (Table 3). Therefore, asymmetry was present in the funnel plot, but it was likely the result of small study effects rather than publication bias.

**TABLE 3:** Effect sizes as a function of the characteristics of the studies (e.g., the design of the study) or interventions (e.g., type of intervention, inclusion of specific behavior change techniques [BCT]) in studies included in the meta‐analysis of the impact of visiting zoos and engaging with zoo‐led interventions on outcomes in zoo visitors.

	*k*	*d*	95% CI	*p*	*p*_subgroup_
**Categorical moderator**					
*Outcome*					0.12
Knowledge	37	0.43	0.31–0.56	<0.001	
Attitudes	30	0.38	0.25–0.50	<0.001	
Self‐efficacy	4	0.35	0.14–0.55	<0.001	
Social norms	0				
Intentions	14	0.44	0.29–0.59	<0.001	
Behavior	7	0.22	0.02–0.43	0.03	
*Type of intervention*					0.23
Digital	3	0.39	−0.09 to 0.88	0.11	
Exhibit design	7	0.07	−0.23 to 0.37	0.66	
Formal education	9	0.57	0.30–0.84	<0.001	
General visit	12	0.44	0.21–0.67	<0.001	
Keeper talk	9	0.35	0.12–0.57	0.01	
Live animal interaction	12	0.43	0.23–0.63	<0.001	
Multimedia	1				
School field trip	2				
Signage	2				
Other	1				
*BCT 1.9: commitment*					0.45
Present	5	0.26	−0.13 to 0.65	0.20	
Not present	51	0.41	0.29–0.53	<0.001	
*BCT 5.3: information about social and environmental consequences*					0.75
Present	22	0.42	0.24–0.60	<0.001	
Not present	35	0.39	0.24–0.53	<0.001	
*BCT 6.1: demonstration of the behavior*					
Present	1				
Not present	55				
*BCT 8.1: behavioral practice or rehearsal*					
Present	1				
Not present	55				
*BCT 9.1: credible source*					0.51
Present	12	0.33	0.11–0.55	0.01	
Not present	45	0.41	0.29–0.54	<0.001	
*BCT 12.5: adding objects to the environment*					0.95
Present	5	0.41	0.02–0.81	0.04	
Not present	51	0.40	0.28–0.52	<0.001	
*Location*					0.14
Aquarium	3	0.58	0.07–1.10	0.03	
Zoo	44	0.40	0.27–0.52	<0.001	
Zoo and aquarium	6	0.14	−0.19 to 0.47	0.39	
Other	3	0.83	0.32–1.33	0.01	
*Region*					0.13
Asia	2				
Australia	8	0.32	0.04–0.61	0.03	
Europe	19	0.55	0.36–0.74	<0.001	
North America	22	0.28	0.10–0.45	<0.001	
South America	2				
Multiple	3	0.16	−0.30 to 0.63	0.49	
*Study design*					0.13
Cohort	23	0.54	0.36–0.71	<0.001	
Cohort analytic	16	0.30	0.09–0.51	0.01	
Controlled comparison	17	0.30	0.09–0.51	0.01	
*Participant design* ^a^					<0.01
Between participants	40	0.30	0.17–0.43	<0.001	
Within participants	16	0.64	0.43–0.84	<0.001	
*Type of data*					0.16
Qualitative	4	0.46	0.03–0.88	0.04	
Quantitative	36	0.32	0.17–0.46	<0.001	
Mixed	16	0.56	0.35–0.77	<0.001	
*Type of sample*					0.22
Adult	36	0.31	0.17–0.45	<0.001	
Child	3	0.42	−0.04 to 0.87	0.07	
School	15	0.59	0.37–0.81	<0.001	
Mixed	3	0.47	−0.01 to 0.95	0.05	
*Publication status*					0.92
Published	41	0.40	0.26–0.53	<0.001	
Not published	15	0.41	0.18–0.64	<0.001	
*Theory mentioned*					0.78
Yes	21	0.42	0.23–0.61	<0.001	
No	35	0.39	0.24–0.53	<0.001	
*Collaborations* ^a^					0.03
Research at multiple institutions	10	0.14	−0.12 to 0.40	0.29	
Research at a single institution	46	0.46	0.33–0.58	<0.001	
*Effect measurement*					0.07
Immediate	53	0.41	0.30–0.53	<0.001	
Delayed	4	0.18	−0.08 to 0.44	0.16	
**Continuous moderator**					
*Year*	56				0.68
*Age of participants*	21				0.30
*Female participants (%)* ^a^	37				0.02

Abbreviations: BCT, behavior change techniques; CI, confidence interval.

^a^ Significant moderators of the pooled effect size estimate in subgroup analysis.

### Moderators of the effect of visiting and engaging with zoos on outcomes

On average, interventions involving formal education (*d*_+_ = 0.57) had a large effect. General visits (*d*_+_ = 0.44) and live animal interactions (*d*_+_ = 0.43) had small to medium effects. Interventions that involved changing exhibit designs had a small effect on outcomes (*d*_+_ = 0.07) (Table 3). The nature of the intervention did not have a significant effect on the overall pooled effect size estimate (*p* > 0.05); thus, the different types of interventions had equivalent positive effects on outcomes (Table 3).

There were sufficient studies to evaluate the impact of 4 BCTs on outcomes in the subgroup analyses. Interventions, including BCT 1.9 commitment (*d*_+_ = 0.26) and BCT 9.1 credible source (*d*_+_ = 0.33), had small effects, whereas BCT 5.3 information about social and environmental consequences (*d*_+_ = 0.42) and BCT 12.5 adding objects to the environment (*d*_+_ = 0.41) had small to medium effects.

Zoo‐led interventions typically had a medium effect on knowledge (*d*_+_ = 0.43) and intentions (*d*_+_ = 0.44), a small to medium effect on attitudes (*d*_+_ = 0.38) and self‐efficacy (*d*_+_ = 0.35), and a small effect on behavior (*d*_+_ = 0.22). The nature of the outcome did not have a significant effect on the overall pooled effect size estimate (*p* > 0.05); thus, visiting and engaging with zoos had equivalent positive effects on the different outcomes (Table 3).

Three variables significantly influenced the effect of zoo‐led interventions on outcomes: design of the study, whether the research was conducted at multiple institutions, and percentage of females in the sample (Table 3). Specifically, studies with within‐participant designs (*d*_+_ = 0.64) typically reported larger effects than studies with between‐participants designs (*d*_+_ = 0.30). Research conducted at a single institution typically reported larger effects (*d*_+_ = 0.46) than research conducted at multiple institutions (*d*_+_ = 0.14). Finally, studies with samples containing a higher percentage of females typically reported smaller effects than studies with a smaller percentage of females (*p* < 0.05).

The remaining study characteristics did not significantly moderate the pooled estimates (Table 3). For instance, effect sizes in studies that mentioned theory did not differ significantly from effect sizes in studies that did not mention theory (*p* > 0.05) (Table 3). Finally, no significant differences were found between effects calculated immediately after engaging with interventions relative to effects calculated with data collected after a delay (*p* > 0.05) (Table 3).

## DISCUSSION

We analyzed 56 studies that evaluated the effects of a range of zoo‐led interventions on visitors’ beliefs about conservation and associated behaviors and found that visiting and engaging with zoos had a positive impact on outcomes, including knowledge about conservation issues, attitudes toward conservation, visitors’ self‐efficacy, intentions to act, and conservation behavior. These findings support other meta‐analyses that suggest conservation education can affect outcomes that may influence conservation behavior, such as beliefs about conservation that we included in our review (Barragan‐Jason et al., 2023; van de Wetering et al., 2022; Whitburn et al., 2020). Taken together, these findings attest to the potential of zoos to promote conservation and shift beliefs in zoo visitors. These shifts may contribute to achieving global targets (Moss et al., 2023) and serve as part of strategic conservation plans (Greenwell et al., 2023; Thomas, 2020).

Conservation education, included in some of the zoo‐led interventions in the present review, is typically used to raise awareness about a specific conservation issue, often in the hope that people will be subsequently more likely to act for the benefit of biodiversity (Schilbert & Scheersoi, 2023; Thomas et al., 2019). Although we focused on engagements in zoos, aquariums, and other locations, it is likely that similar approaches are utilized in other institutions or contexts in which conservation education is delivered (e.g., museums, by environmental nongovernmental organizations). For instance, a science festival in Australia sought to communicate conservation ideas, such as adopting more sustainable business practices and greening one's home (Mair & Laing, 2013). A systematic review of global conservation education programs shows that evaluations would benefit from a more holistic approach to measuring the success of such programs (Thomas et al., 2019). Conservation educators or institutions should see evaluating the outcomes of their work as an opportunity to prove and share their real‐world impacts, which would enable them—and others—to draw on evidence when designing future interventions (Krasny, 2020). Our hope is that our findings will help build a structured evidence base on which researchers and practitioners, particularly those looking to adopt structured approaches to designing and evaluating behavioral interventions that have proved valuable in other disciplines (e.g., the behavior change wheel [Michie et al., 2011]), can develop and evaluate interventions in a variety of contexts.

Our results illustrate the breadth of ways visitors can engage with zoos and the scope of strategies used in the provision of conservation education in zoos. To date, 6 specific BCTs have been used in interventions to change behavior in zoo visitors. Three of these BCTs were relatively common (i.e., increasing awareness of behaviors and their impacts [BCT 5.3], overcoming barriers to action [BCT 12.5], and delivering information from a trusted source [BCT 9.1]). The 6 techniques we identified make up a small subset of the potential number of techniques available. There are over 90 techniques according to the BCT, and the newly published revised version describes over 250 techniques (Michie et al., 2013, 2021). A plethora of strategies are therefore available for zoo practitioners and researchers to use as components of interventions. Indeed, including a wider range of strategies (e.g., BCTs) could potentially help zoo practitioners increase their behavior change impact (Barongi et al., 2015).

Although the primary interventions we reviewed used a range of approaches, on average, all types of interventions we identified had positive impacts on outcomes in zoo visitors. One interpretation of these findings is that a visit or any form of engagement with a zoo or aquarium is likely to have positive effects on outcomes in visitors. However, effect sizes were variable among studies, and there was insufficient evidence to estimate the impact of some types of interventions. Therefore, further evaluations of specific intervention approaches are warranted to address the gaps in the evidence we identified. This review demonstrates that theoretical frameworks from behavioral science (e.g., the behavior change wheel [ Michie et al., 2011]) can provide a systematic way to investigate the impact of engaging with zoos and aid practitioners in developing interventions in response to research findings (Krasny, 2020; Maynard et al., 2021). Going forward, the use of mixed‐methods approaches to assessing intervention impact, accommodating both qualitative and quantitative data analysis, might also provide more nuanced insights (Atkins et al., 2017; Thomas et al., 2019; Tuite, 2022). Practitioners and researchers might use this methodological framework to accumulate the evidence base in the field.

### Impact of zoo‐led interventions on measured outcomes

**FIGURE 5:**
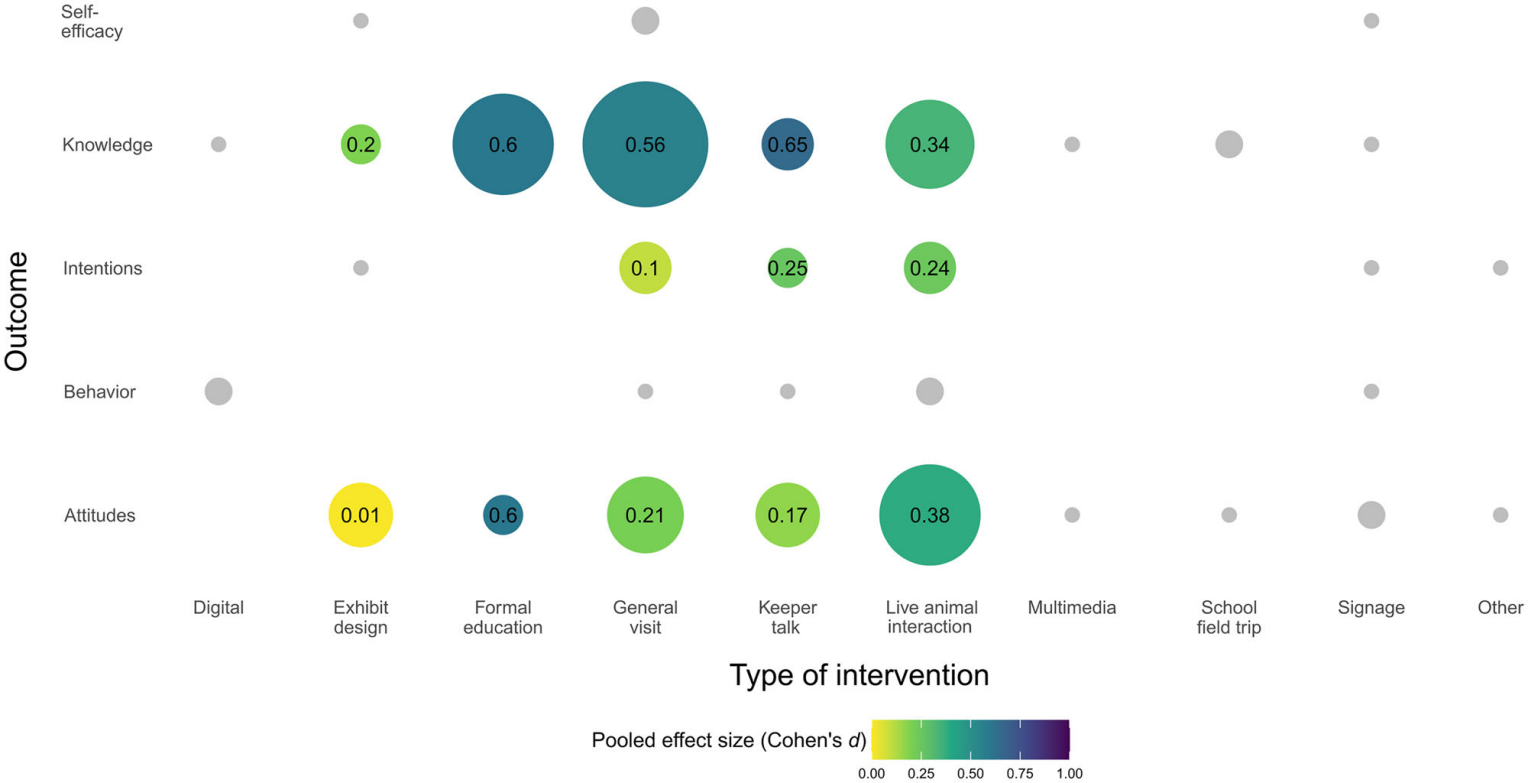
Number of studies (circle size proportional to number of studies) evaluating the effect of different types of interventions on outcomes in the meta‐analysis of the impact of visiting zoos and engaging with zoo‐led interventions on outcomes in visitors and the pooled effect size (circle color according to pooled effect of each type of intervention on each outcome) (gray circles, combinations of identified interventions and outcomes with <3 studies for which sample‐weighted average effects were not computed).

The majority of primary studies in the present review focused on the effect of interventions on visitors’ knowledge or attitudes (Table 3) (Figure 5). Outcomes such as self‐efficacy and intentions were measured in fewer studies, and social norms were not evaluated in any studies, despite suggestions that these outcomes can reliably predict and drive behavior (Ajzen, 1991; Armitage & Conner, 2001). Given the often‐cited gap between knowledge and behavior (Knutti, 2019; van de Watering et al., 2022), researchers and practitioners should seek to supplement measures of knowledge or attitudes with measures of beliefs that are associated with behavior. For instance, outcomes described by the TPB in this review (e.g., intentions, self‐efficacy) that show positive effects in response to zoo‐led interventions can be included in future evaluations. Further, considering the moral or personal obligations of conservation behavior, research may benefit from including measures of social norms (De Groot & Steg, 2009; Harland et al., 1999). It is encouraging to find that zoo‐led interventions had a positive effect on all outcomes because research in clinical contexts typically shows that interventions have significantly larger effects on knowledge or attitudes than on intentions or behavior (e.g., Trivasse et al., 2020). Our findings, however, suggest that visiting and engaging with zoos can have a positive effect on visitors’ knowledge, beliefs, and behavior. This conclusion should be taken in the context that, to date, zoos have typically assessed a limited range of outcomes in the research (Moss et al., 2023; Nygren & Ojalammi, 2018; Spooner et al., 2023; Thomas, 2020).

### Considerations for future research

Our results identify a number of problems to address. First, for research to be useful for researchers and practitioners alike, the components of interventions (e.g., BCTs or mode of delivery) and the methodologies of evaluations need to be reported clearly. We found that many zoo‐led interventions are described without the necessary clarity for meaningful replication—an observation that mirrors discussions in the field of health psychology prior to the development of theoretical frameworks such as those we used here (Michie & Johnston, 2012; Michie et al., 2009). For example, a review evaluating nondrug interventions claims that around 60% of research describes the intervention in adequate detail (Hoffman et al., 2013). In response, guidelines for reporting, such as the TIDieR checklist, have been published (Hoffman et al., 2014). We advocate the use of such frameworks in zoo‐led research to make them easier to compare and aggregate in research synthesis. Specifically, the TIDieR framework suggests that researchers report effect size metrics alongside each analysis or complete information needed to compute effect sizes. We also suggest that interventions be described with reference to the specific BCTs used (Michie et al., 2013, 2021), the outcomes the techniques targeted (Schenk et al., 2023), the modes by which each technique was delivered (Marques et al., 2021), and whether theory was used and, if so, how (Michie & Prestwich, 2010).

Second, we recommend that researchers and practitioners measure proximal determinants of behavior as specified by theoretical frameworks in addition to (or even instead of) changes in knowledge, which is a relatively poor predictor of behavior (Knutti, 2019). We used the TPB (Ajzen, 1991) as a framework for identifying and classifying the outcomes measured in the primary research. We found no evidence of the effects of perceptions of social norms, despite reviews of the evidence suggesting that people's beliefs about significant others’ attitudes toward behavior or others’ actual behavior are considered reliable determinants of behavior with respect to environmental protection and conservation (Farrow et al., 2017; Yamin et al., 2019). This is one example of how future zoo‐led research can utilize evidence to measure (and explicitly target) outcomes, such as social norms, with the evidence base from fields such as behavioral science.

Third, the effects of moderating variables warrant discussion with respect to future research. We identified larger effects in within‐participant designs that measured changes in beliefs in the same individuals over time, relative to between‐participant designs that assessed differences between participants (e.g., those exposed vs. not exposed to an intervention). This disparity was likely driven by reduced variability in within‐participant designs; examining the same participants across conditions can allow researchers to control for individual differences in their study design. This boosts statistical power, making it easier to detect effects relative to between‐participant designs, where different participants introduce variability. Studies evaluating interventions in a single institution demonstrated larger effects than those across multiple zoological institutions. Using this approach also minimizes variability. By delivering and evaluating interventions in a single location, researchers can control for the context in which the intervention was delivered. Although within‐participant designs and single‐context studies can yield effects with reduced variability and increased control, the generalizability of the findings is restricted. We recommend that future researchers use methods that allow them to control for specific extraneous variables that may affect impacts in different contexts. For instance, intervention fidelity measures can be implemented to assess the actual delivery in each context compared with the planned objectives (Knittle, 2014; McKenna et al., 2014). Conceptual replication studies, especially those spanning multiple contexts, can offer insights into the global impact of zoo‐led interventions, but it is imperative that they capture comprehensive details about intervention delivery, audience, and potential moderating variables.

Fourth, the extent to which the effects of zoo‐led interventions on outcomes are durable should be considered in future. To facilitate comparison between the interventions, we calculated effects across short periods in over 90% of studies in this review, which leaves open the possibility that the reported effects may not be durable beyond this period. Our results showed that this seems unlikely because no significant differences were found among studies evaluating immediate effects relative to delayed effects and because our sensitivity analyses, which included effects calculated using data from the longest follow‐up point, rather than shortest, showed that effects (*d*_+_ = 0.38) were similar to those of the primary analyses (*d*_+_ = 0.40). This suggests that effects may be durable over time, but more research is needed to understand the longer term durability of impacts on visitors’ beliefs.

### Limitations

Our study had several limitations. First, there was limited evidence available for specific types of study characteristics, zoo‐led interventions, and outcomes. For example, as demonstrated in the evidence‐gap map (Figure 5), there is a paucity of research on interventions that utilize multimedia or digital approaches. Therefore, we were unable to include these interventions in the moderator analyses. Second, the search strategy was limited to the chosen sources in this review. However, we are confident that the search conducted in Web of Science was sufficiently comprehensive. Furthermore, the impacts of engaging with zoos that we found may not be representative of all zoos; the majority of studies were situated in 3 industrialized countries (i.e., United Kingdom, United States, and Australia), and, although no language restrictions were placed on the search, only one of the included studies was written in a language other than English (Rato, 2020).

Taken together, our findings make important strides in bringing the evidence on the effect of zoo‐led interventions together. The meta‐analysis revealed that a visit to the zoo or engagement with zoo‐led interventions positively affects multiple outcomes in zoo visitors; people left the zoo with more knowledge about conservation issues and more favorable attitudes toward conservation and reported a greater likelihood to act for the benefit of biodiversity. Our hope is that these findings—together with the methodological framework for characterizing the nature of interventions and outcomes—provide a foundation for further research in this area that can build cumulative evidence on the impact of zoos and aquariums on those who visit and engage with them and, by extension, efforts to conserve and promote biodiversity.

### OPEN RESEARCH BADGES

This article has earned Open Data, Open Materials and Preregistered Research Design badges. Data and materials are available at https://osf.io/dtz6q/files/osfstorage and Preregistered is available at https://osf.io/wnh2b.

## Supporting information

Supporting Information

Supporting Information

Supporting Information

Supporting Information

Supporting Information
