# Supplementary material for: A meta‐analysis of the effect of visiting zoos and aquariums on visitors’ conservation knowledge, beliefs, and behavior
Source: Conserv Biol. 2024 Feb 2;39(1):e14237. doi: 10.1111/cobi.14237 (PMC11780219; doi:10.1111/cobi.14237)
Supplement: Supplementary file 1 — Supporting Information [file COBI-39-e14237-s004.docx]

**Search terms**

The search terms are as follows:

*Web of Science:*

The search conducted in Web of Science used the field tag “TS=”, this searches the title, abstracts and keywords of the articles.

TS=

((zoo OR zoos OR aquarium OR aquaria OR “safari park” OR “wildlife park” OR “nature reserve” OR “wildlife sanctuary”)

**AND**

(psychosocial OR psychology OR knowledge OR attitude* OR belief* OR intention* OR empower* OR behaviour* OR behavior* OR connection)

**AND**

(education OR intervention OR visit* OR engagement OR experience))

*ProQuest:*

The search conducted in ProQuest used the field code “TI,AB:”, this searches the title and abstract of the articles.

TI,AB:

((zoo OR zoos OR aquarium OR aquaria OR “safari park” OR “wildlife park” OR “nature reserve” OR “wildlife sanctuary”)

**AND**

(psychosocial OR psychology OR knowledge OR attitude* OR belief* OR intention* OR empower* OR behaviour* OR behavior* OR connection)

**AND**

(education OR intervention OR visit* OR engagement OR experience))
