# Supplementary material for: A meta‐analysis of the effect of visiting zoos and aquariums on visitors’ conservation knowledge, beliefs, and behavior
Source: Conserv Biol. 2024 Feb 2;39(1):e14237. doi: 10.1111/cobi.14237 (PMC11780219; doi:10.1111/cobi.14237)
Supplement: Supplementary file 2 — Supporting Information [file COBI-39-e14237-s003.docx]

**Coding manual**

The studies included in the review utilise a variety of study designs and report their findings differently. The following principles describe the rationale for the decisions made when coding in this review:

*Coding interventions*

- Research articles with both between-participant and within-participant designs are included in this review, with between-participant comparisons prioritised when extracting information from studies. Effect sizes generated from within-participant designs need to consider the dependency between samples collected from the same participants and as such, they are influenced by characteristics in the sampling strategy (Cuijpers et al., 2017).
- In this review, the meta-analysis will be unable to account for interaction effects and therefore a single comparison was selected from studies with this type of design. Research articles that use study designs with multiple comparison groups compared through interactions add complexity to the analyses.
- Intention-to-treat (ITT) designs were preferenced over per-protocol analyses as ITT designs allow researchers to maintain planned sampling strategies and control for biases effectively (Gupta, 2011). In this research context, a comparison that investigated the presence of an intervention would be preferred rather than a design that considered whether zoo visitors chose to engage with an intervention.
- Participant exposure to an intervention/treatment should be clearly defined and measured. If a research article determined participant exposure to interventions via retrospective self-reported survey items, then alternative outcomes with clear immediate measurements were coded or the research article was excluded from the analysis.
- One study reported data for pre and post-measurements made up of matched and unmatched samples combined. This creates a complex dependence structure in the data as some comparisons are correlated. The author was contacted and data for the unmatched sample only was requested, this was used for the analysis.
- The measurement of the shortest duration was coded for each comparison, for example, a comparison that measured outcomes prior to an engagement with an intervention and following an engagement. If follow-up measurements were reported in this example, these were not used to calculate effect sizes (N.B., except in the sensitivity analysis).

*Calculating effect sizes*

- The protocol for gathering effect sizes from the research articles prioritised effect size values reported by the author and then the use of online tools to calculate effect sizes. When calculating the effect sizes, raw data (e.g., mean, standard deviation) was used in the first instance. If sufficient raw data was not reported then summary statistics (e.g., F value reported in the output of ANOVA analysis) were used.
- To calculate the effect sizes and variances for each comparison, the reported sample sizes of each condition were used in the calculations. For articles that used a single control group and multiple intervention/treatment groups; the sample size of the control group was divided by the total number of intervention/treatment groups to calculate the variance.

**Effect size calculations**

The studies included in the review utilise a variety of study designs and report their findings differently. The following principles describe the process of calculating effect sizes in this review:

*Between participant designs*

- Tool 1: Used to convert Means and SD for groups with equal sample size to Cohen’s d. The groups have the same sample size, the effect size is calculated by subtracting the means and dividing the result by the pooled standard deviation.
- Tool 2: Used to convert Means and SD for groups with different sample sizes to Cohen’s d. The effect size is computed by adjusting the calculation of the pooled standard deviation with weights for sample sizes.
- Tool 5: Used to convert independent t-test statistics to Cohen’s d, based on formulas reported by Borenstein (2009, p.28).
- Tool 6: Used to convert F-value of ANOVA and sample size to Cohen’s d. The calculation only works for ANOVAs with two distinct groups (Thalheimer & Cook, 2002).
- Tool 11: Used to convert non-parametric test statistics (e.g., Mann-Whitney U, Kruskal-Wallis H) to Cohen’s d.
- Tool 14: Used to convert effect sizes reported by the study authors to Cohen’s d (e.g., convert an eta squared effect size to Cohen’s d)
- Tool 15: Used to convert chi-squared X^2^ value to Cohen’s d. Chi squared calculated by creating contingency tables for binary outcome variables (e.g., Yes/No, Correct/Incorrect).

*Within participant designs*

- Change Score Standardisation: Used to convert Mean & SD reported for each measure (Cohen, 1988; Lakens, 2013)
- Tool 5: Used to convert dependent t-test statistics to Cohen’s d, although this calculator does not consider the dependency in the t value. It is preferable to use Change Score Standardisation where data is available.
- Tool 11: Used to convert non-parametric test statistics (e.g., Mann-Whitney U, Kruskal-Wallis H) to Cohen’s d.
- Tool 14: Used to convert effect sizes reported by the study authors to Cohen’s d (e.g., convert an eta squared effect size to Cohen’s d)

**Variance Calculations**

*Between participant designs*

The variance formula will use the sample sizes and effect size to calculate a variance for Cohen's d (Borenstein et al., 2009, Cooper et al., 2009).

*Within participant designs*

The variance formula will use the sample size, effect size and correlation between scores to calculate a variance for Cohen's d (Borenstein et al. 2009, Gibbons et al., 1993).
