# Supplementary material for: A meta‐analysis of the effect of visiting zoos and aquariums on visitors’ conservation knowledge, beliefs, and behavior
Source: Conserv Biol. 2024 Feb 2;39(1):e14237. doi: 10.1111/cobi.14237 (PMC11780219; doi:10.1111/cobi.14237)
Supplement: Supplementary file 4 — Supporting Information [file COBI-39-e14237-s002.docx]

**README: Multi-Level Meta-Analysis & Evidence Gap Map (EGM) in R**

Table of Contents

1. Overview

2. Prerequisites

3. Instructions

- Multi-Level Meta-Analysis

- Evidence Gap Map

4. Code Structure

5. Notes

6. License

Overview

This R script performs a multi-level meta-analysis on the provided data set. It starts by setting up the working environment, then loads the necessary data, conducts a multi-level meta-analysis, validates this model, assigns weights, and visualizes the results through funnel and forest plots. The provided code details a method to visualize gaps in the research literature concerning specific interventions and outcomes using an evidence gap map. It uses various packages and custom functions to achieve this, offering a clear visual summary of the state of the research.

This repository contains two distinct R scripts:

1. Multi-Level Meta-Analysis: Performs a comprehensive three-level meta-analysis on the provided data, including model validation, variance attribution, weight calculation, and result visualization through funnel and forest plots.

2. Evidence Gap Map (EGM): Creates a visual representation indicating the concentration of studies concerning specific interventions and outcomes within a meta-analysis context.

Prerequisites

Software Requirement - RStudio or R environment

**Dependencies**

You need to install the following R packages:

- For Multi-Level Meta-Analysis: `metafor`, `ggplot2`, `devtools`, `dmetar` (from GitHub).

- For EGM: `tidyverse`, `janitor`, `metafor`, `clubSandwich`.

This code is written to analyse the specific provided dataset.

- The variable structure is outlined below

**Variable structure**

**Study** {text - character}: describing the study citation, i.e., authors and publication year

**Author** {text - character]: describing study author

**Count** {numeric}: counting variable for each study

**Comparison** {numeric}: counting variable for each comparison

**Year** {numeric}: describing the publication year of source

**Publication** {text - character}: describing the publication status of the source

**Pub_pub** {numeric}: describing the publication status of the source, i.e., 1=published, 0=unpublished

**Pub_unpub** {numeric}: describing the publication status of the source, i.e., 1=unpublished, 0=published

**Search_Source** {text - character}: describing the search strategy source, i.e., Web of Science, ProQuest, grey literature

**Source** {text - character}: describing the source of the study i.e., journal, thesis, grey literature

**Language** {text - character}: describing the written language of the study, i.e., EN=English, PT=Portuguese

**Study_Design** {text - character}: describing the study design (in reference to EPHPP tool)

**Subjects** {text - character}: describing the study design, i.e., B=between participant, W=within participant

**Data_Type** {text - character}: describing the type of data collected, i.e., Quan=quantitative data, Qual=qualitative data, Mixed=mixed methods

**Method** {text - character}: describing the method used to collect data

**Method_Report** {text - character}: describing whether the data collection method was reported, i.e., with sufficient detail to allow replication

**Impact_duration** {text - character}: describing the time period of the effect size calculation, i.e., Immediate, Delayed

**Impact_time** {numeric}: describing the time (in weeks) that data was measured

**Follow_Up** {text-character}: describing the presence of a follow up measurement, i.e., Yes, No

**FU_Time** {numeric}: describing the time (in weeks) of the longest follow up present

**Age** {numeric}: describing the average age of the sample

**Female** {numeric}: describing the percentage of female participants in the sample

**Sample_Type** {text - character}: describing the type of sample, i.e., Adult=over 18 years of age, Child=less than 18 years of age (and not a school group), School=less than 18 years of age and with a school group, Other=mixed sample of adults and children

**Region** [text - character}: describing the region in which data was collected

**Country** {text - character}: describing the country in which data was collected, i.e., AU=Australia, BR=Brazil, CA=Canada, CN=China, DE=Germany, FR=France, GB=United Kingdom, GT=Guatemala, IE=Ireland, MULTI=Multiple countries, NZ=New Zealand, PT=Portugal, US=United States, VN=Vietnam

**Collaboration** {text - character}: describing whether the study was conducted at multiple institutions or a single institution

**Zoo_Aqua** {text - character}: describing the name of the institution in which the study was conducted (MULTI=study conducted at multiple institutions

**Intervention** {text - character}: describing the type of intervention (Other=type of intervention not captured in descriptions)

**Where** {text - character}: describing where the intervention was delivered, i.e., Aqua=aquarium, Zoo=zoo, ZooAqua=combination of zoos and aquariums, Other=location other than zoo or aquarium

**Theory_Type** {text - character}: describing the type of theory mentioned

**Theory** {text - character}: describing whether theory was mentioned

**BCT 1.9** {text - character}: describing whether BCT 1.9 was present

**BCT 5.3** {text - character}: describing whether BCT 5.3 was present

**BCT 6.1** {text - character}: describing whether BCT 6.1 was present

**BCT 8.1** {text - character}: describing whether BCT 8.1 was present

**BCT 9.1** {text - character}: describing whether BCT 9.1 was present

**BCT 12.5** {text - character}: describing whether BCT 12.5 was present

**Outcome** {text - character}: describing the outcome measured

**Measure** {text - character}: describing the type of measure used to collect data, i.e., Close=close ended questions, Likert=Likert scale, Open=open ended questions, Other=measure described as other (e.g., connectedness to nature scale)

**Reliability** {text - character}: describing measures of reliability for scale measures, i.e., Cron=Cronbach’s alpha, KMO= Kaiser Meyer Olkin, Pear=Pearsons, RHO= reliability rho, None=no reliability reported

**Value** {numeric}: describing the value given for the reliability for scale measures

**nTm** {numeric}: describing the sample size of the treatment group

**nCc** {numeric}: describing the sample size for the control/comparison group

**nTotal** {numeric}: describing the total sample size of the study

**ES_Report** {text - character}: describing whether the effect size was reported by the study author or calculated, i.e., Calc=effect size calculated, Report=effect size reported

**ES_d** {numeric}: describing the effect size value (Cohen’s d_+_)

**VAR_d** {numeric}: describing the variance of the effect size

**es.id** {numeric}: counting variable for each effect size

**Tool** {numeric}: describing the tool/method used to calculate effect sizes i.e., 1=Psychometrica Tool 1, 2=Psychometrica Tool 2, 3=Psychometrica Tool 3, 5=Psychometrica Tool 5, 6=Psychometrica Tool 6, 11=Psychometrica Tool 11, 14=Psychometrica Tool 14, 15=Psychometrica Tool 15, RM1=change score standardisation, 0=effect size reported

Instructions

Multi-Level Meta-Analysis

1. **Environment Setup**: Clear the R environment and load the required packages.

2. **Data Loading**: Replace placeholders like `"Working Directory"` and `"File Name.csv"` with your specific directory and filename.

3. **Run the Script**: Execute the multi-level meta-analysis script. Ensure the column names in the data match `ES_d` and `VAR_d`.

4. **Outputs**: Results include:

- ANOVA table for model comparison.

- Funnel plot saved as 'Contour enhanced funnel plot - aggregated.png'.

- Forest plot saved as 'Forest plot - with number of studies (ki).png'.

Evidence Gap Map

1. **Data Preparation**: Ensure the CSV format and the presence of columns "Intervention", "Outcome", "ES_d", "VAR_d", "Study".

2. **Configuration**: Update the `read.table()` function with the appropriate file path.

3. **Run the Script**: Execute the EGM script.

4. **Outputs**: A PNG file named `"egm_plotggsave.png"` saved in the working directory showcasing the EGM.

Code Structure

Multi-Level Meta-Analysis

1. **Data Loading and Environment Setup**: Includes loading libraries and the data file.

2. **Multi-Level Meta-Analysis Execution**: Performs the 3-level meta-analysis.

3. **Model Validation**: Uses ANOVA for model comparisons.

4. **Result Visualisation**: Produces funnel and forest plots.

Evidence Gap Map

1. **Loading and Cleaning Data**: Includes data reading and column name standardization.

2. **Creating Summary Data**: Processes the data and performs a multivariate meta-analysis, if applicable.

3. **EGM Plot Creation**: Constructs a scatter plot indicating the number of studies and pooled effect size.

Notes

**Redundancies**: Ensure to review redundant operations, such as placeholder values like, "Working Directory", "File Name.csv" and "XX.XX".

**Customisations**: Adjust file paths, column names, and visualization settings to match your dataset and preferences.

**Data Back-up**: Always keep an original data backup before script execution.

License

Scripts are provided "as is" without warranties or guarantees. You're free to modify, distribute, and use them as you see fit.

End of README.

## clear environment ------------------------------------------------

rm(list=ls())

## install & load packages ------------------------------------------

install.packages("metafor")

library(metafor)

install.packages("ggplot2")

library(ggplot2)

if (!require("devtools")) {

install.packages("devtools")}

devtools::install_github("MathiasHarrer/dmetar")

library(dmetar)

##`````` set working directory & load file --------------------------

#--- edit this accordingly

setwd("Working Directory")

mydata <- read.csv("File Name.csv")

##`````` Multi-Level Meta-analysis ----------------------------------

# 3 level model

mlmodel1 <- rma.mv(yi = ES_d,

V = VAR_d,

data = mydata,

random = ~ 1 | Study/es.id,

test = "t",

method = "REML")

summary(mlmodel1)

predict(mlmodel1)

confint(mlmodel1)

##`````` Validate Multi-level Meta-analysis -------------------------

# ANOVA to compare 2 level with 3 level model

m_within_null <- rma.mv(yi = ES_d,

V = VAR_d,

data = mydata,

random = ~ 1 | Study/es.id,

sigma2 = c(0, NA))

m_between_null <- rma.mv(yi = ES_d,

V = VAR_d,

data = mydata,

random = ~ 1 | Study/es.id,

sigma2 = c(NA, 0))

m_both_null <- rma.mv(yi = ES_d,

V = VAR_d,

data = mydata,

random = ~ 1 | Study/es.id,

sigma2 = c(0, 0))

aov_within <- anova(mlmodel1, m_within_null)

aov_between <- anova(mlmodel1, m_between_null)

aov_bothnull <- anova(mlmodel1, m_both_null)

# Join these results in a table

aov_table <- rbind(

c(df=aov_between$p.f, aov_between$fit.stats.f[c(3:4, 1)],

LRT = NA, p = NA),

c(df=aov_within$p.r, aov_within$fit.stats.r[c(3:4, 1)],

LRT = aov_within$LRT, p = aov_within$pval),

c(df=aov_between$p.r, aov_between$fit.stats.r[c(3:4, 1)],

LRT = aov_between$LRT, p = aov_between$pval),

c(df=aov_bothnull$p.r, aov_bothnull$fit.stats.r[c(3:4, 1)],

LRT = aov_bothnull$LRT, p = aov_bothnull$pval)

)

rownames(aov_table) <- c("Three-level model",

"Within-studies variance constrained",

"Between-studies variance constrained",

"Both variance components constrained")

aov_table

# Look at AIC and BIC values, and significance of LRT

##`````` Attribution of variance ----------------------------------

#### ATTRIBUTION of variance ####

# need to install {metar} base code to use var.comp

# copy and paste the below into the console and hit ENTER

mlm.variance.distribution = var.comp = function(x){

m = x

# Check class

if (!(class(m)[1] %in% c("rma.mv", "rma"))){

stop("x must be of class 'rma.mv'.")

}

# Check for three level model

if (m$sigma2s != 2){

stop("The model you provided does not seem to be a three-level model. This function can only be used for three-level models.")

}

# Check for right specification (nested model)

if (sum(grepl("/", as.character(m$random[[1]]))) < 1){

stop("Model must contain nested random effects. Did you use the '~ 1 | cluster/effect-within-cluster' notation in 'random'? See ?metafor::rma.mv for more details.")

}

# Get variance diagonal and calculate total variance

n = m$k.eff

vector.inv.var = 1/(diag(m$V))

sum.inv.var = sum(vector.inv.var)

sum.sq.inv.var = (sum.inv.var)^2

vector.inv.var.sq = 1/(diag(m$V)^2)

sum.inv.var.sq = sum(vector.inv.var.sq)

num = (n-1)*sum.inv.var

den = sum.sq.inv.var - sum.inv.var.sq

est.samp.var = num/den

# Calculate variance proportions

level1=((est.samp.var)/(m$sigma2[1]+m$sigma2[2]+est.samp.var)*100)

level2=((m$sigma2[2])/(m$sigma2[1]+m$sigma2[2]+est.samp.var)*100)

level3=((m$sigma2[1])/(m$sigma2[1]+m$sigma2[2]+est.samp.var)*100)

# Prepare df for return

Level=c("Level 1", "Level 2", "Level 3")

Variance=c(level1, level2, level3)

df.res=data.frame(Variance)

colnames(df.res) = c("% of total variance")

rownames(df.res) = Level

I2 = c("---", round(Variance[2:3], 2))

df.res = as.data.frame(cbind(df.res, I2))

totalI2 = Variance[2] + Variance[3]

# Generate plot

df1 = data.frame("Level" = c("Sampling Error", "Total Heterogeneity"),

"Variance" = c(df.res[1,1], df.res[2,1]+df.res[3,1]),

"Type" = rep(1,2))

df2 = data.frame("Level" = rownames(df.res),

"Variance" = df.res[,1],

"Type" = rep(2,3))

df = as.data.frame(rbind(df1, df2))

g = ggplot(df, aes(fill=Level, y=Variance, x=as.factor(Type))) +

coord_cartesian(ylim = c(0,1), clip = "off") +

geom_bar(stat="identity", position="fill", width = 1, color="black") +

scale_y_continuous(labels = scales::percent)+

theme(axis.title.x=element_blank(),

axis.text.y = element_text(color="black"),

axis.line.y = element_blank(),

axis.title.y=element_blank(),

axis.line.x = element_blank(),

axis.ticks.x = element_blank(),

axis.text.x = element_blank(),

axis.ticks.y = element_line(lineend = "round"),

legend.position = "none",

panel.grid.major = element_blank(),

panel.grid.minor = element_blank(),

panel.background = element_blank(),

legend.background = element_rect(linetype="solid",

colour ="black"),

legend.title = element_blank(),

legend.key.size = unit(0.75,"cm"),

axis.ticks.length=unit(.25, "cm"),

plot.margin = unit(c(1,3,1,1), "lines")) +

scale_fill_manual(values = c("darkseagreen3", "deepskyblue3", "darkseagreen2",

"deepskyblue1", "deepskyblue2")) +

# Add Annotation

# Total Variance

annotate("text", x = 1.5, y = 1.05,

label = paste("Total Variance:",

round(m$sigma2[1]+m$sigma2[2]+est.samp.var, 3))) +

# Sampling Error

annotate("text", x = 1, y = (df[1,2]/2+df[2,2])/100,

label = paste("Sampling Error Variance: \n", round(est.samp.var, 3)), size = 3) +

# Total I2

annotate("text", x = 1, y = ((df[2,2])/100)/2-0.02,

label = bquote("Total"~italic(I)^2*":"~.(round(df[2,2],2))*"%"), size = 3) +

annotate("text", x = 1, y = ((df[2,2])/100)/2+0.05,

label = paste("Variance not attributable \n to sampling error: \n", round(m$sigma2[1]+m$sigma2[2],3)), size = 3) +

# Level 1

annotate("text", x = 2, y = (df[1,2]/2+df[2,2])/100, label = paste("Level 1: \n",

round(df$Variance[3],2), "%", sep=""), size = 3) +

# Level 2

annotate("text", x = 2, y = (df[5,2]+(df[4,2]/2))/100,

label = bquote(italic(I)[Level2]^2*":"~.(round(df[4,2],2))*"%"), size = 3) +

# Level 3

annotate("text", x = 2, y = (df[5,2]/2)/100,

label = bquote(italic(I)[Level3]^2*":"~.(round(df[5,2],2))*"%"), size = 3)

returnlist = list(results = df.res,

totalI2 = totalI2,

plot = g)

class(returnlist) = c("mlm.variance.distribution", "list")

invisible(returnlist)

returnlist

}

i2 <- var.comp(mlmodel1)

i2

##`````` Weights --------------------------------------------------

# check study weights in the model given to each study

wi.all <- weights(mlmodel1, type="rowsum")

sum(wi.all * mydata$ES_d) / sum(wi.all)

weight.all <- data.frame(k = c(table(mydata$Study)),

weight = tapply(wi.all, mydata$Study, sum))

# open weight to see the list and sort by weights

# studies with most effect sizes have most weight

# assess if studies are in order of contributed effect sizes (k)

##`````` Funnel plot ----------------------------------------------

# Plot funnel with effect sizes aggregated to study level

# create an 'escalc' object (and add study labels)

dat1 <- escalc(measure="SMD", yi=ES_d, vi=VAR_d, data=mydata)

V1 <- vcalc(VAR_d, cluster=Study, data=dat1, rho=0.6)

### fit multilevel model using this approximate V matrix

res1 <- rma.mv(ES_d, diag(V1),

random = ~ 1 | Study/es.id,

data=dat1,

method="REML")

agg1 <- aggregate(dat1, cluster=Study, V=vcov(res1, type="obs"), addk=TRUE)

agg1.1 <- agg1[c(1,50,51,52)]

# N.B. Variables: 1=Study

# 50=yi (effect sizes)

# 51=vi (variance)

# 52=ki (number of estimates)

rescheck1 <- rma(yi, vi, method="EE", data=agg1.1)

rescheck1

png(file='Contour enhanced funnel plot - aggregated.png',

units='cm',

width=30, height=20,

res=300)

funnel_agg <- funnel(rescheck1, level=c(90, 95, 99),

yaxis="vi",

shade=c("white", "gray55", "gray75"),

legend=TRUE,

at=seq(-1,2.5,by=0.5),

refline=0,

xlab="Cohen's d")

dev.off()

# funnel plot saves to desktop

##`````` Forest plot ----------------------------------------------

#create function to add text for model results

mlabfun1 <- function(text, x, y) {

list(bquote(paste(.(text),

" (Q = ", .(formatC(x$QE, digits=2, format="f")),

", df = ", .(x$k - x$p),

", p ", .(metafor:::.pval(x$QEp, digits=2, showeq=TRUE, sep=" ")), "; ",

I^2, " = ", .(formatC("XX.XX%)")))))} # insert i2 variance

# forest plot with ki, CI

png(file='Forest plot - with number of studies (ki).png',

units='cm',

width=35, height=30,

res=300)

forest_agg <- forest(rescheck1, xlim=c(-2,3.75), ylim=c(-2,59), alim=c(-1,3),

cex=0.8,

header=TRUE,

ilab=ki, ilab.xpos=-1.1,

xlab="Pooled effect size (Cohen's d)",

slab=agg1$Study,

order="obs",

at=seq(-1,3.5,by=0.5),

border="black",

col="grey",

mlab=mlabfun1("RE Model", mlmodel1, i2_1))

text(-1.1, rescheck1$k+2, "Estimates", cex=0.8, col="black", font=2)

dev.off()

# forest plot saves to desktop

##`````` Test for publication bias --------------------------------

# Egger's test

mlmodelpubbias <- rma.mv(yi = ES_d,

V = VAR_d,

data = mydata,

random = ~ 1 | Study/es.id,

mods = ~ VAR_d,

test = "t",

method = "REML")

mlmodelpubbias

# "Egger's" test with variance as predictor

# assess intercept value significance

# Assink & Wibbelink - dummy variable for publication status

mydata$Pub_pub <- as.factor(mydata$Pub_pub)

mydata$Pub_unpub <- as.factor(mydata$Pub_unpub)

notpublished <- rma.mv(yi = ES_d,

V = VAR_d,

data = mydata,

random = ~ 1 | Study/es.id,

mods = ~ Pub_pub,

test = "t",

method = "REML")

summary(notpublished, digits=3) # unpublished are reference

# gives estimate for unpublished studies

published <- rma.mv(yi = ES_d,

V = VAR_d,

data = mydata,

random = ~ 1 | Study/es.id,

mods = ~ Pub_unpub,

test = "t",

method = "REML")

summary(published, digits=3) # published are reference

# gives estimate for published studies

## Test of mods - assess significance

##`````` Outliers - influence analysis -----------------------------

# Cook's Distance analysis

xall <- cooks.distance(mlmodel1, progbar=TRUE, cluster=Study,

reestimate=TRUE, parallel="no", ncpus=1)

png(file='Cooks Distance Plot.png')

cooksplot_all <- plot(xall, type="o", pch=19, xlab="Observed Outcome",

ylab="Cook's Distance",

xaxt="n")

axis(side=1, at=seq_along(xall), labels=as.numeric(names(xall)))

dev.off()

# identify influential cases

# output returns influential cases according to rule

# - 4/n threshold

xallinfluential <- xall[xall>(4/56)]

xallinfluential

# - rule of thumb not > 3*Mean

xallinfluential_mean <- xall[xall>(3*mean(xall))]

xallinfluential_mean

##`````` Sensitivity analysis - outliers removed -------------

mydata_fin <- subset(mydata,

Study!="Outlier 1" &

Study!="Outlier 2" &

Study!="Outlier 3" &

Study!="Outlier 4")

## Multi-Level Meta-analysis ---------------------------------

# 3 level model

mlmodel_fin <- rma.mv(yi = ES_d,

V = VAR_d,

data = mydata_fin,

random = ~ 1 | Study/es.id,

method="REML",

test="t")

summary(mlmodel_fin)

predict(mlmodel_fin)

confint(mlmodel_fin)

## Attribution of Variance -----------------------------------

i2.out <- var.comp(mlmodel_fin)

i2.out

##`````` Sensitivity analysis - longest time effect measurement -------------

# read in data file with effects calculated over longest time periods

mydata_long <- read.csv("File Name.csv")

## Multi-Level Meta-analysis ---------------------------------

# 3 level model

mlmodel_long <- rma.mv(yi = ES_d,

V = VAR_d,

data = mydata_long,

random = ~ 1 | Study/es.id,

method="REML",

test="t")

summary(mlmodel_long)

predict(mlmodel_long)

confint(mlmodel_long)

##`````` Subgroup analyses ----------------------------------------

# Categorical Predictors

### Outcome ###

#check out if Outcome significant moderator

mlmodel_out <- rma.mv(ES_d,

VAR_d,

data = mydata,

random = ~ 1 | Study/es.id,

mods= ~ Outcome,

test = "t",

method = "REML")

mlmodel_out

#Attitudes as reference

mlmodel_out.0 <- rma.mv(ES_d,

VAR_d,

data = mydata,

random = ~ 1 | Study/es.id,

mods= ~ 0 + Outcome,

test = "t",

method = "REML")

mlmodel_out.0

### Intervention ###

# recoded to remove subgroups with less than 3 studies

mydata_int <- subset(mydata, Intervention!="School field trip" &

Intervention!="Multimedia" & Intervention!="Other" &

Intervention!="Signage")

#check out if Intervention significant moderator

mlmodel_int <- rma.mv(ES_d,

VAR_d,

data = mydata_int,

random = ~ 1 | Study/es.id,

mods= ~ Intervention,

test = "t",

method = "REML")

mlmodel_int

#Digital as reference

mlmodel_int.0 <- rma.mv(ES_d,

VAR_d,

data = mydata_int,

random = ~ 1 | Study/es.id,

mods= ~ 0 + Intervention,

test = "t",

method = "REML")

mlmodel_int.0

### BCT 1.9 ###

#check out if BCT 1.9 significant moderator

mlmodel_BCT1.9 <- rma.mv(ES_d,

VAR_d,

data = mydata,

random = ~ 1 | Study/es.id,

mods= ~ BCT1.9,

test = "t",

method = "REML")

mlmodel_BCT1.9

mlmodel_BCT1.9.0 <- rma.mv(ES_d,

VAR_d,

data = mydata,

random = ~ 1 | Study/es.id,

mods= ~ 0 + BCT1.9,

test = "t",

method = "REML")

mlmodel_BCT1.9.0

### BCT 5.3 ###

#check out if BCT 5.3 significant moderator

mlmodel_BCT5.3 <- rma.mv(ES_d,

VAR_d,

data = mydata,

random = ~ 1 | Study/es.id,

mods= ~ BCT5.3,

test = "t",

method = "REML")

mlmodel_BCT5.3

mlmodel_BCT5.3.0 <- rma.mv(ES_d,

VAR_d,

data = mydata,

random = ~ 1 | Study/es.id,

mods= ~ 0 + BCT5.3,

test = "t",

method = "REML")

mlmodel_BCT5.3.0

### BCT 9.1 ###

#check out if BCT 9.1 significant moderator

mlmodel_BCT9.1 <- rma.mv(ES_d,

VAR_d,

data = mydata,

random = ~ 1 | Study/es.id,

mods= ~ BCT9.1,

test = "t",

method = "REML")

mlmodel_BCT9.1

mlmodel_BCT9.1.0 <- rma.mv(ES_d,

VAR_d,

data = mydata,

random = ~ 1 | Study/es.id,

mods= ~ 0 + BCT9.1,

test = "t",

method = "REML")

mlmodel_BCT9.1.0

### BCT 12.5 ###

#check out if BCT 12.5 significant moderator

mlmodel_BCT12.5 <- rma.mv(ES_d,

VAR_d,

data = mydata,

random = ~ 1 | Study/es.id,

mods= ~ BCT12.5,

test = "t",

method = "REML")

mlmodel_BCT12.5

mlmodel_BCT12.5.0 <- rma.mv(ES_d,

VAR_d,

data = mydata,

random = ~ 1 | Study/es.id,

mods= ~ 0 + BCT12.5,

test = "t",

method = "REML")

mlmodel_BCT12.5.0

### Location ###

#check out if Location significant moderator

mlmodel_loc <- rma.mv(ES_d,

VAR_d,

data = mydata,

random = ~ 1 | Study/es.id,

mods= ~ Where,

test = "t",

method = "REML")

mlmodel_loc

mlmodel_loc.0 <- rma.mv(ES_d,

VAR_d,

data = mydata,

random = ~ 1 | Study/es.id,

mods= ~ 0 + Where,

test = "t",

method = "REML")

mlmodel_loc.0

### Regions ###

# recoded to remove groups with less than 3 studies

mydata_reg <- subset(mydata, Region!="Asia" &

Region!="South America")

#check out if Region significant moderator

mlmodel_reg <- rma.mv(ES_d,

VAR_d,

data = mydata_reg,

random = ~ 1 | Study/es.id,

mods= ~ Region,

test = "t",

method = "REML")

mlmodel_reg

mlmodel_reg.0 <- rma.mv(ES_d,

VAR_d,

data = mydata_reg,

random = ~ 1 | Study/es.id,

mods= ~ 0 + Region,

test = "t",

method = "REML")

mlmodel_reg.0

### Study Design ###

#check out if Study Design significant moderator

mlmodel_stud <- rma.mv(ES_d,

VAR_d,

data = mydata,

random = ~ 1 | Study/es.id,

mods= ~ Study_Design,

test = "t",

method = "REML")

mlmodel_stud

mlmodel_stud.0 <- rma.mv(ES_d,

VAR_d,

data = mydata,

random = ~ 1 | Study/es.id,

mods= ~ 0 + Study_Design,

test = "t",

method = "REML")

mlmodel_stud.0

### Participant Structure ###

#check out if participant structure significant moderator

mlmodel_sub <- rma.mv(ES_d,

VAR_d,

data = mydata,

random = ~ 1 | Study/es.id,

mods= ~ Subjects,

test = "t",

method = "REML")

mlmodel_sub

mlmodel_sub.0 <- rma.mv(ES_d,

VAR_d,

data = mydata,

random = ~ 1 | Study/es.id,

mods= ~ 0 + Subjects,

test = "t",

method = "REML")

mlmodel_sub.0

### Data Type ###

#check out if Data Type significant moderator

mlmodel_data <- rma.mv(ES_d,

VAR_d,

data = mydata,

random = ~ 1 | Study/es.id,

mods= ~ Data_Type,

test = "t",

method = "REML")

mlmodel_data

mlmodel_data.0 <- rma.mv(ES_d,

VAR_d,

data = mydata,

random = ~ 1 | Study/es.id,

mods= ~ 0 + Data_Type,

test = "t",

method = "REML")

mlmodel_data.0

### Sample Type ###

#check out if Sample Type significant moderator

mlmodel_sam <- rma.mv(ES_d,

VAR_d,

data = mydata,

random = ~ 1 | Study/es.id,

mods= ~ Sample_Type,

test = "t",

method = "REML")

mlmodel_sam

mlmodel_sam.0 <- rma.mv(ES_d,

VAR_d,

data = mydata,

random = ~ 1 | Study/es.id,

mods= ~ 0 + Sample_Type,

test = "t",

method = "REML")

mlmodel_sam.0

### Theory ###

#check out if Theory significant moderator

mlmodel_Theory <- rma.mv(ES_d,

VAR_d,

data = mydata,

random = ~ 1 | Study/es.id,

mods= ~ Theory,

test = "t",

method = "REML")

mlmodel_Theory

mlmodel_Theory.0 <- rma.mv(ES_d,

VAR_d,

data = mydata,

random = ~ 1 | Study/es.id,

mods= ~ 0 + Theory,

test = "t",

method = "REML")

mlmodel_Theory.0

### Collaborations ###

#check out if Collaborations significant moderator

mlmodel_coll <- rma.mv(ES_d,

VAR_d,

data = mydata,

random = ~ 1 | Study/es.id,

mods= ~ Collaboration,

test = "t",

method = "REML")

mlmodel_coll

mlmodel_coll.0 <- rma.mv(ES_d,

VAR_d,

data = mydata,

random = ~ 1 | Study/es.id,

mods= ~ 0 + Collaboration,

test = "t",

method = "REML")

mlmodel_coll.0

### Effect measurement time ###

#check out if effect measurement significant moderator

mlmodel_ESmeasure <- rma.mv(ES_d,

VAR_d,

data = mydata,

random = ~ 1 | Study/es.id,

mods= ~ Impact_duration,

test = "t",

method = "REML")

mlmodel_ESmeasure

mlmodel_ESmeasure.0 <- rma.mv(ES_d,

VAR_d,

data = mydata,

random = ~ 1 | Study/es.id,

mods= ~ 0 + Impact_duration,

test = "t",

method = "REML")

mlmodel_ESmeasure.0

# Continuous predictors

### Age ###

# recoded to remove studies with no age reported, Age=0

mydata_age <- subset(mydata, Age>1)

mlmodel_age <- rma.mv(ES_d,

VAR_d,

data = mydata_age,

random = ~ 1 | Study/es.id,

mods= ~ Age,

test = "t",

method = "REML")

mlmodel_age

### Year ###

mlmodel_year <- rma.mv(ES_d,

VAR_d,

data = mydata,

random = ~ 1 | Study/es.id,

mods= ~ Year,

test = "t",

method = "REML")

mlmodel_year

### %Female ###

# recoded to remove studies with no %Female reported

mydata_fem <- subset(mydata, Female>1)

mlmodel_fem <- rma.mv(ES_d,

VAR_d,

data = mydata_fem,

random = ~ 1 | Study/es.id,

mods= ~ Female,

test = "t",

method = "REML")

mlmodel_fem

##`````` Evidence Gap Map ------------------------------------

# Load packages

library(tidyverse)

library(janitor)

library(clubSandwich)

# Load data and clean -----------------------------------------

dat <-

read.table("File Name.csv", header = TRUE, sep = ",",

stringsAsFactors = FALSE) %>%

clean_names(case = "parsed")

dat <- dat[, c("Intervention", "Outcome", "ES_d", "VAR_d", "Study")]

names(dat) <- c("factor_1", "factor_2", "es", "var", "study_id")

# Create Summary Data -----------------------------------------

tidy_meta <- function(dat){

summary <- dat %>%

summarize(n_studies = n_distinct(study_id),

n_es = n())

m <- summary %>% pull(n_studies)

n <- summary %>% pull(n_es)

if(m > 2){

mod <- rma.mv(es,

var,

data = dat,

random = ~ 1 | study_id/es,

test = "t",

method = "REML")

res <- conf_int(mod, vcov = "CR2", tidy = TRUE) %>%

rename(estimate = beta) %>%

as_tibble() %>%

mutate(method = "Correlated Effects")

} else{

res <- tibble(estimate = NA,

SE = NA,

df = NA,

CI_L = NA,

CI_U = NA,

method = "Less then 3 studies")

}

output <- bind_cols(res, summary) %>%

mutate_if(is.numeric, round, 3) %>%

select(method, estimate, n_studies, n_es)

return(output)

}

summary_dat <-

dat %>%

group_by(factor_1, factor_2) %>%

group_modify(~ tidy_meta(.x)) %>%

ungroup()

# Create EGM plot ---------------------------------------------

p <- ggplot(summary_dat, aes(x = factor_1, y = factor_2, size = n_studies*2.5,

color = estimate)) +

geom_point(alpha = 1) +

scale_color_viridis_c(na.value="grey", limits = c(0, 1), direction = -1) +

labs(x = "Type of intervention", y = "Outcome measured",

color = "Pooled effect size (Cohen's d)", size=3) +

scale_size_identity() +

scale_x_discrete(labels = function(x) str_wrap(x, width = 10)) +

scale_y_discrete(labels = function(x) str_wrap(x, width = 10)) +

theme_minimal() +

guides(size = "none") +

theme(legend.position = "bottom", axis.text=element_text(size=8),

axis.title=element_text(size=9),

panel.grid.major = ggplot2::element_blank(),

panel.grid.minor = ggplot2::element_blank(),

panel.background = element_rect(fill = "white", colour = "white",

size = 2, linetype = "solid"),

plot.background = element_rect(fill = "white"),

legend.title = element_text(size=7),

legend.text = element_text(size=6))

p <- p +

geom_text(aes(label = as.character(round(estimate, 2))),

size = 3, color = "black")

p

ggsave(plot = p, filename="egm_plotggsave.png")
