# Supplementary material for: A meta‐analysis of the effect of visiting zoos and aquariums on visitors’ conservation knowledge, beliefs, and behavior
Source: Conserv Biol. 2024 Feb 2;39(1):e14237. doi: 10.1111/cobi.14237 (PMC11780219; doi:10.1111/cobi.14237)
Supplement: Supplementary file 5 — Supporting Information [file COBI-39-e14237-s005.docx]

Anderson, U., Kelling, A., Pressley-Keough, R., Bloomsmith, M., & Maple, T. (2003). Enhancing the zoo visitor's experience by public animal training and oral interpretation at an otter exhibit. *Environment and Behavior, 35*(6), 826-841.

Ballantyne, R., Packer, J., Hughes, K., & Gil, C. (2018). Post-Visit Reinforcement of Zoo Conservation Messages: The Design and Testing of an Action Resource Website. *Visitor Studies, 21*(1), 98-120.

Bueddefeld, J., & Van Winkle, C. (2017). Exploring the effect of zoo post-visit action resources on sustainable behavior change. *Journal of Sustainable Tourism, 25*(9), 1205-1221.

Carlin, K. (1999). *The impact of curiosity on learning during a school field trip to the zoo* (Publication Number 9945941) [Ph.D., University of Florida]. ProQuest Dissertations and Theses Global.

Chalmin-Pui, L., & Perkins, R. (2017). How do visitors relate to biodiversity conservation? An analysis of London Zoo's "BUGS' exhibit. *Environmental Education Research, 23*(10), 1462-1475.

Chiew, S., Hemsworth, P., Sherwen, S., Melfi, V., & Coleman, G. (2019). The Effect of Regulating Zoo Visitor-Penguin Interactions on Zoo Visitor Attitudes. *Frontiers in Psychology, 10*, https://doi.org/10.3389/fpsyg.2019.02351

Chung, T., Wilsey, S., Mykita, A., Lesgold, E., & Bourne, J. (2019). Quick response code scanning for children's informal learning. *International Journal of Information and Learning Technology, 36*(1), 38-51.

Clayton, S., Bexell, S., Ping, X., Zhihe, Z., Jing, L., Wei, C., & Yan, H. (2018). Confronting the wildlife trade through public education at zoological institutions in Chengdu, PR China. *Zoo Biology, 37*(2), 119-129.

Clayton, S., Prevot, A., Germain, L., & Saint-Jalme, M. (2017). Public Support for Biodiversity After a Zoo Visit: Environmental Concern, Conservation Knowledge, and Self-Efficacy. *Curator, 60*(1), 87-100.

Collins, C., Corkery, I., McKeown, S., McSweeney, L., Flannery, K., Kennedy, D., & O'Riordan, R. (2020). Quantifying the long-term impact of zoological education: a study of learning in a zoo and an aquarium. *Environmental Education Research, 26*(7), 1008-1026.

Craig, L., & Vick, S. (2021). Engaging Zoo Visitors at Chimpanzee (Pan troglodytes) Exhibits Promotes Positive Attitudes Toward Chimpanzees and Conservation. *Anthrözoos, 34*(1), 1-15.

da Silva, M., Braga-Pereira, F., da Silva, M., de Oliveira, J., Lopes, S., & Alves, R. (2021). What are the factors influencing the aversion of students towards reptiles? *Journal of Ethnobiology and Ethnomedicine, 17*(1). https://doi.org/10.1186/s13002-021-00462-z

Falk, J., & Adelman, L. (2003). Investigating the impact of prior knowledge and interest on aquarium visitor learning. *Journal of Research in Science Teaching, 40*(2), 163-176.

Geiger, N., Swim, J., Fraser, J., & Flinner, K. (2017). Catalyzing Public Engagement With Climate Change Through Informal Science Learning Centers. *Science Communication, 39*(2), 221-249.

Herendeen, T. (2017). *Comparing the Reactions of Zoo Visitors to Immersive and Nonimmersive Exhibit Experiences* (Publication Number 10760185) [M.S., East Carolina University]. ProQuest Dissertations and Theses Global.

Jacobson, S., Hopper, L., Shender, M., Ross, S., Leahy, M., & McNernie, J. (2017). Zoo visitors' perceptions of chimpanzee welfare are not affected by the provision of artificial environmental enrichment devices in a naturalistic exhibit. *Journal of Zoo and Aquarium Research, 5*(1), 56-61.

Jensen, E. (2014). Evaluating Children's Conservation Biology Learning at the Zoo. *Conservation Biology, 28*(4), 1004-1011.

Kelly, A., & Skibins, J. (2021). Inspiring Wildlife Conservation Behaviors through Innovations in Zoo Exhibit Design. *Visitor Studies, 24*(1), 79-99.

Kim Ho, L., Vu, P., & Nguyen, N. (2018). Valuing Nature in Childhood. *International Zoo Educators Journal, 54*, 13-16.

Kirchgessner, M. (2014). *The impact of zoo live animal presentations on students' propensity to engage in conservation behaviors* (Publication Number 3671924) [Ed.D., Temple University]. ProQuest Dissertations and Theses Global.

Kleespies, M., Gubert, J., Popp, A., Hartmann, N., Dietz, C., Spengler, T., Becker, M., & Dierkes, P. (2020). Connecting High School Students With Nature - How Different Guided Tours in the Zoo Influence the Success of Extracurricular Educational Programs. *Frontiers in Psychology, 11*. https://doi.org/10.3389/fpsyg.2020.01804

Lakes, R. (2016). *An examination of intrinsic existence value towards wildlife of Columbus Zoo and Aquariums tourists: Evaluating the impact of behind the scenes programming* (Publication Number 10112009) [D.E., Eastern Kentucky University]. ProQuest Dissertations and Theses Global.

Liu, B. (2017). Evaluating students’ learning from a zoo based unit of work. *International Zoo Educators Journal, 53*, 6-9.

Lukas, K., & Ross, S. (2014). Naturalistic Exhibits May be More Effective Than Traditional Exhibits at Improving Zoo-Visitor Attitudes toward African Apes. *Anthrözoos, 27*(3), 435-455.

MacDonald, E. (2015). Quantifying the Impact of Wellington Zoo's Persuasive Communication Campaign on Post-Visit Behavior. *Zoo Biology, 34*(2), 163-169.

Mallavarapu, S., & Taglialatela, L. (2019). A post-occupancy evaluation of the impact of exhibit changes on conservation knowledge, attitudes, and behavior of zoo visitors. *Environmental Education Research, 25*(10), 1552-1569.

McLeod, E., & Rawson, S. (2019). Lord Howe Island Stick Insect Encounters. *International Zoo Educators Journal, 55*, 44-49.

Mellish, S., Pearson, E., McLeod, E., Tuckey, M., & Ryan, J. (2019). What goes up must come down: an evaluation of a zoo conservation-education program for balloon litter on visitor understanding, attitudes, and behaviour. *Journal of Sustainable Tourism, 27*(9), 1393-1415.

Mellish, S., Sanders, B., Litchfield, C., & Pearson, E. (2017). An investigation of the impact of Melbourne Zoo's "Seal-the-Loop" donate call-to-action on visitor satisfaction and behavior. *Zoo Biology, 36*(3), 237-242.

Miller, L., Luebke, J., Matiasek, J., Granger, D., Razal, C., Brooks, H., & Maas, K. (2020). The impact of in-person and video-recorded animal experiences on zoo visitors' cognition, affect, empathic concern, and conservation intent. *Zoo Biology, 39*(6), 367-373.

Miller, L., Zeigler-Hill, V., Mellen, J., Koeppel, J., Greer, T., & Kuczaj, S. (2013). Dolphin Shows and Interaction Programs: Benefits for Conservation Education? *Zoo Biology, 32*(1), 45-53.

Moss, A., Jensen, E., & Gusset, M. (2015). Evaluating the contribution of zoos and aquariums to Aichi Biodiversity Target 1. *Conservation Biology, 29*(2), 537-544.

Moss, A., Jensen, E., & Gusset, M. (2017). Impact of a global biodiversity education campaign on zoo and aquarium visitors. *Frontiers in Ecology and the Environment, 15*(5), 243-247.

Pavitt, B., & Moss, A. (2019). Assessing the effect of zoo exhibit design on visitor engagement and attitudes towards conservation. *Journal of Zoo and Aquarium Research, 7*(4), 186-194.

Pearson, E., Lowry, R., Dorrian, J. & Litchfield, C. (2014). Evaluating the Conservation Impact of an Innovative Zoo-Based Educational Campaign: 'Don't Palm Us Off' for Orang-utan Conservation. *Zoo Biology, 33*(3), 184-196.

Price, E., Ashmore, L., & McGivern, A. (1994). Reactions of zoo visitors to free-range monkeys. *Zoo Biology, 13*(4), 355-373.

Randall, T. (2011). *Assessment of change in conservation attitudes through zoo education* (Publication Number 3468955) [Ph.D., Oklahoma State University]. ProQuest Dissertations and Theses Global.

Randler, C., Kummer, B., & Wilhelm, C. (2012). Adolescent Learning in the Zoo: Embedding a Non-Formal Learning Environment to Teach Formal Aspects of Vertebrate Biology. *Journal of Science Education and Technology, 21(*3), 384-391.

Rato, D. (2020). *Love, Learn, Protect: Assessing the Short-Term Impact of Lisbon Zoo School Education Programs on 10-18 Years Old Students* (Publication Number 28787637) [M.S., Universidade de Lisboa (Portugal)]. ProQuest Dissertations and Theses Global.

Roa, M. (2016). *The influence of caring, curiosity, and knowledge on visitors' conservation intentions: An exploratory investigation at the Tracy Aviary* (Publication Number 10157929) [M.S., The University of Utah]. ProQuest Dissertations and Theses Global.

Roberts, M. (2013). *Mandatory interpretation for coastal protected areas* (Publication Number U620242) [Ph.D., University of Portsmouth (United Kingdom)]. ProQuest Dissertations and Theses Global.

Sampson, L., Riley, J., & Carpenter, A. (2020). Applying IUCN reintroduction guidelines: An effective medium for raising public support prior to conducting a reintroduction project. *Journal for Nature Conservation, 58.* https://doi.org/10.1016/j.jnc.2020.125914

Sattler, S., & Bogner, F. (2017). Short- and long-term outreach at the zoo: cognitive learning about marine ecological and conservational issues. *Environmental Education Research, 23*(2), 252-268.

Sellmann, D., & Bogner, F. (2013). Climate change education: quantitatively assessing the impact of a botanical garden as an informal learning environment. *Environmental Education Research, 19*(4), 415-429.

Skibins, J., & Powell, R. (2013). Conservation Caring: Measuring the Influence of Zoo Visitors' Connection to Wildlife on Pro-Conservation Behaviors. *Zoo Biology, 32*(5), 528-540.

Smart, T., Counsell, G., & Quinnell, R. (2021). The impact of immersive exhibit design on visitor behaviour and learning at Chester Zoo, UK. *Journal of Zoo and Aquarium Research, 9*(3), 139-149.

Spooner, S., Jensen, E., Tracey, L., & Marshall, A. (2019). Evaluating the impacts of theatre-based wildlife and conservation education at the zoo. *Environmental Education Research, 25*(8), 1231-1249.

Spooner, S., Jensen, E., Tracey, L., & Marshall, A. (2021). Evaluating the effectiveness of live animal shows at delivering information to zoo audiences. *International Journal of Science Education Part B-Communication and Public Engagement, 11*(1), 1-16.

Staus, N. (2012). *Crossing the Cartesian Divide: An Investigation into the Role of Emotion in Science Learning (*Publication Number 3514854) [Ph.D., Oregon State University]. ProQuest Dissertations and Theses Global.

Syrowicz, V. (2018). Sustainability Education as a Zoo Exhibit. *International Zoo Educators Journal, 54,* 45-47.

Torpie-Sweterlitsch, J. (2015). *Captive nature: Exploring the influence of zoos on visitor worldview, knowledge, and behavior* (Publication Number AAI1565202) [M.A., University of Texas]. ProQuest Dissertations and Theses Global.

Visscher, N., Snider, R., & Stoep, G. (2009). Comparative Analysis of Knowledge Gain Between Interpretive and Fact-Only Presentations at an Animal Training Session: An Exploratory Study. *Zoo Biology, 28*(5), 488-495.

Waller, B., Peirce, K., Mitchell, H., & Micheletta, J. (2012). Evidence of Public Engagement with Science: Visitor Learning at a Zoo-Housed Primate Research Centre. *Plos One, 7*(9). https://doi.org/10.1371/journal.pone.0044680

Walsh, L. (2015). *Learning from a dive show in an aquarium setting* (Publication Number 1595797) [M.S., California State University, Long Beach]. ProQuest Dissertations and Theses Global.

Whitehouse, J., Waller, B., Chanvin, M., Wallace, E., Schel, A., Peirce, K., Mitchell, H., Macri, A., & Slocombe, K. (2014). Evaluation of Public Engagement Activities to Promote Science in a Zoo Environment. *Plos One, 9*(11). https://doi.org/10.1371/journal.pone.0113395

Wunschmann, S., Wust-Ackermann, P., Randler, C., Vollmer, C., & Itzek-Greulich, H. (2017). Learning Achievement and Motivation in an Out-of-School Setting-Visiting Amphibians and Reptiles in a Zoo Is More Effective than a Lesson at School. *Research in Science Education, 47*(3), 497-518.
